# Enhanced classification performance using deep learning based segmentation for pulmonary embolism detection in CT angiography

**DOI:** 10.1016/j.heliyon.2024.e38118

**Published:** 2024-09-19

**Authors:** Ali Teymur Kahraman, Tomas Fröding, Dimitris Toumpanakis, Christian Jamtheim Gustafsson, Tobias Sjöblom

**Affiliations:** a Department of Immunology, Genetics and Pathology, Uppsala University, Uppsala, Sweden; b Department of Radiology, Nyköping Hospital, Nyköping, Sweden; c Karolinska University Hospital, Stockholm, Sweden; d Department of Surgical Sciences, Uppsala University, Sweden; e Department of Hematology Oncology and Radiation Physics, Skåne University Hospital, Lund, Sweden; f Department of Translational Sciences, Medical Radiation Physics, Lund University, Malmö, Sweden

**Keywords:** Computed tomography pulmonary angiography, Pulmonary embolism, nnU-net, Deep learning

## Abstract

**Purpose:**

To develop a deep learning-based algorithm that automatically and accurately classifies patients as either having pulmonary emboli or not in CT pulmonary angiography (CTPA) examinations.

**Materials and methods:**

For model development, 700 CTPA examinations from 652 patients performed at a single institution were used, of which 149 examinations contained 1497 PE traced by radiologists. The nnU-Net deep learning-based segmentation framework was trained using 5-fold cross-validation. To enhance classification, we applied logical rules based on PE volume and probability thresholds. External model evaluation was performed in 770 and 34 CTPAs from two independent datasets.

**Results:**

A total of 1483 CTPA examinations were evaluated. In internal cross-validation and test set, the trained model correctly classified 123 of 128 examinations as positive for PE (sensitivity 96.1 %; 95 % C.I. 91–98 %; P < .05) and 521 of 551 as negative (specificity 94.6 %; 95 % C.I. 92–96 %; P < .05), achieving an area under the receiver operating characteristic (AUROC) of 96.4 % (95 % C.I. 79–99 %; P < .05). In the first external test dataset, the trained model correctly classified 31 of 32 examinations as positive (sensitivity 96.9 %; 95 % C.I. 84–99 %; P < .05) and 2 of 2 as negative (specificity 100 %; 95 % C.I. 34–100 %; P < .05), achieving an AUROC of 98.6 % (95 % C.I. 83–100 %; P < .05). In the second external test dataset, the trained model correctly classified 379 of 385 examinations as positive (sensitivity 98.4 %; 95 % C.I. 97–99 %; P < .05) and 346 of 385 as negative (specificity 89.9 %; 95 % C.I. 86–93 %; P < .05), achieving an AUROC of 98.5 % (95 % C.I. 83–100 %; P < .05).

**Conclusion:**

Our automatic pipeline achieved beyond state-of-the-art diagnostic performance of PE in CTPA using nnU-Net for segmentation and volume- and probability-based post-processing for classification.

## 1 Introduction

Pulmonary embolism (PE) is a potentially life-threatening occlusion of pulmonary arteries caused by blood clotting and is associated with significant morbidity and mortality [1]. PE affects >400,000 patients in Europe [2] and between 300,000 and 600,000 patients in the US [3] causing an estimated >100,000 deaths annually [4]. PE is a significant cause of preventable hospital deaths in the world [5], demanding rapid clinical management [6]. The computed tomography pulmonary angiography (CTPA) imaging modality is the current gold standard for PE diagnosis [7]. The CTPA is a CT scan performed after intravenous injection of iodinated contrast medium. As the emboli do not absorb contrast medium they can be recognized as dark filling defects in the pulmonary arteries [8]. Thoroughly examining every CT slice and identification of PE in CTPA is time-consuming for the radiologist and requires considerable training and attentiveness, and the inter-observer variability is high for small, sub-segmental emboli [9]. An automated solution for detection of PE in CTPA has potential to assist the radiologist by reducing reading times and the risk of emboli being overlooked.

Developing a general solution for automatic detection of PE has proven challenging because of anatomical variation, motion and breathing artifacts, inter-patient variability in contrast medium concentration, and concurrent pathologies. Over the past two decades, automated PE detection has been attempted using deterministic models, such as image processing and analysis techniques [10,11], or probabilistic/statistical models such as machine learning [[12], [13], [14]] and deep convolutional neural networks [15,16]. Yet, the accuracies of these solutions have been insufficient for clinical use due to low sensitivity [10,13,15] and high false positive rate [10,11,13,14], potentially caused by training on small datasets [10,11,[13], [14], [15]]. The state-of-the-art is a residual neural network (ResNet) classification architecture on 1465 CTPA examinations with sensitivity of 92.7 % and specificity of 95.5 % [17]. To mitigate the limited dataset size challenges hampering the training of AI models for PE classification, an alternative approach is to employ a fine-tuned U-Net-like semantic segmentation model. The U-Net model has demonstrated its effectiveness in several medical image segmentation tasks [18]. The no-new U-Net framework (nnU-Net) successfully addresses challenges of finding the best U-net model and fine-tuning its hyperparameters [19].

Here, we sought to take advantage of the segmentation performance of the nnU-Net framework in an algorithm that automatically classifies routine patient CTPA examinations as having PE or not with higher sensitivity and specificity than the current state-of-the-art performance.

## 2 Materials and Methods

### 2.1 Internal dataset

The single-institution (Nyköping Hospital, Sweden) retrospective dataset consisted of 700 non-ECG-gated CTPA examinations performed between 2014 and 2018 (n = 149 positive for PE); 383 CTPA examinations from 353 women (age range 16–97 years; median age 73 years; interquartile range 20 years) and 317 from 299 men (age range 19–100 years; median age 71 years; interquartile range 15 years) [20]. The CTPAs were clinical routine examinations exported in chronological order from a history list in the institution's Picture Archiving and Communication System (PACS). The only disruption in the order were a few inserted time gaps, which allowed for a larger number of CT scanners to be included as new CT scanners were installed during the time period. The CTPAs were acquired on five different CT scanners (Somatom Definition Flash, Siemens Healthcare, Erlangen, Germany; LightSpeed VCT, General Electric (GE) Healthcare Systems, Waukesha, WI, USA; Brilliance 64, Ingenuity Core and Ingenuity CT, Philips Medical Systems, Eindhoven, the Netherlands). As contrast medium, Omnipaque 350 mg I/ml (GE Healthcare Systems, Waukesha, WI, USA) was used. Collection and analysis of CTPA examinations was approved by the Swedish Ethical Review Authority (EPN Uppsala Dnr 2015/023 and 2015/023/1). The CTPA data was anonymized and exported in Digital Imaging and Communications in Medicine (DICOM) format, using a hardware solution (Dicom2USB). The CTPAs were reviewed and annotated using the open-source software Medical Imaging Interaction Toolkit (MITK) [21] by two radiologists (DT and TF) with 6 and 16 years of experience. Each CTPA was annotated by either DT or TF. All blood clots in 149 CTPA examinations positive for PE were manually segmented in axial view, image by image, resulting in 36,471 segmentations.

### 2.2 External datasets

**Fig. 1:**
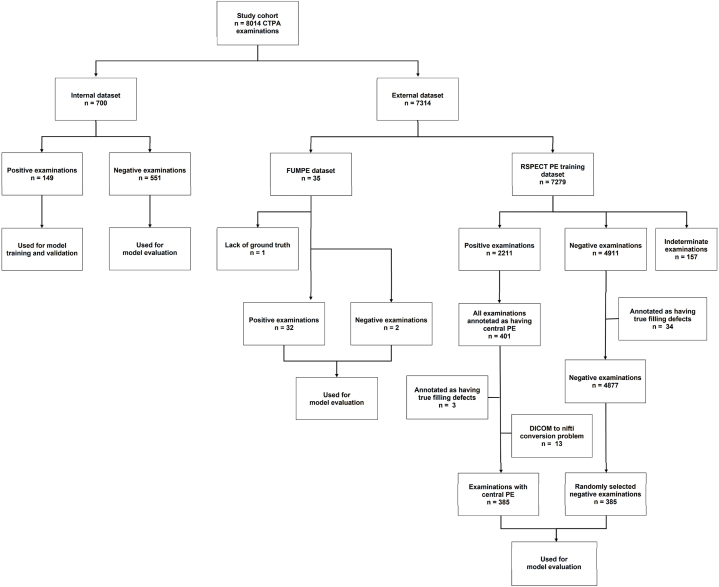
**Internal and external datasets for training and evaluation of a segmentation-based classification model for pulmonary embolism detection.** Positive examinations refer to the patient having pulmonary embolism (PE) and negative examinations are patients without PE. True filling defect refers to tumor invasion, stump thrombus, catheter, embolized wire, or other obvious non-PE condition as defined in the RSPECT dataset.

Two publicly available datasets were used for external evaluation; the Ferdowsi University of Mashhad's PE dataset (FUMPE) [22] and the RSNA-STR Pulmonary Embolism CT (RSPECT) Dataset [23]. The FUMPE dataset contains 35 CTPAs with voxel-level PE annotation by radiologists. Of the 35 CTPAs, two were negative for PE, 32 were positive and one was excluded for lack of ground truth annotation (Supp. materials). The RSPECT dataset consisted of a training (n = 7279) and a test (n = 2167) set and image-level annotations were provided for the training set by several subspecialist thoracic radiologists. 385 CTPAs were selected from the RSPECT training dataset out of a total of 398 that had central PE, and 13 examinations were excluded due to errors during the DICOM to NIfTI format conversion process. Of the 4877 CTPAs without PE or other true filling defect, 385 examinations were randomly selected. An overview of our internal and external datasets is shown in Fig. 1.

### 2.3 nnU-net model training and validation

For model training, the nnU-net DL open-source framework, implemented in a Docker container (Docker Inc., Palo Alto, California, USA), was used [19]. The nnU-Net is a semantic segmentation method, and when provided with a training dataset, it automatically configures an end-to-end experimental pipeline (Supp. materials). The PE positive examinations from the internal dataset (n = 149) were randomly assigned to training (80 %, n = 119) and validation sets (20 %, n = 30) using 5-fold cross-validation during model training (Supp. materials).

### 2.4 Automated classification algorithm

**Fig. 2:**
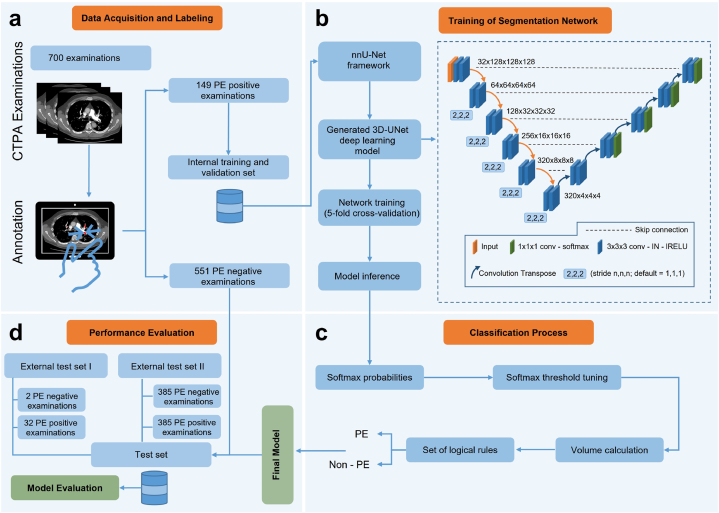
**Training and evaluation of a segmentation-based classification model for pulmonary embolism detection.** 700 CTPA examinations were collected and annotated by either of two radiologists. Of these, all 149 PE positive examinations were used for training and the PE negative were kept for later evaluation (a). The 3D U-Net deep learning model, which is generated by the nnU-Net framework, was trained with the 149 PE-positive CTPAs using 5-fold cross-validation. The convolution layer used a 3 × 3 × 3 filter size by default, followed by an instance normalization (IN) layer and a leaky rectified Linear Unit (lRELU) layer (b). The *softmax* probabilities were obtained from the model inference for fine-tuning classification into PE or non-PE voxel classes and for calculating the predicted PE volume. By thresholding the predicted volumes and applying a set of logical rules, accurate patient-level classification for PE was achieved (c). The final model was evaluated on 804 external CTPA examinations from two publicly available datasets (d).

After model training and validation, the validated model was embedded in a classification algorithm consisting of three steps, pre-processing, image segmentation inference, and post-processing (Fig. 2). Notably, nnU-Net necessitates the utilization of the Neuroimaging Informatics Technology Initiative (NIfTI) file format for model inference. Thus, in the pre-processing step, all DICOM data were converted to the NIfTI format using an in-house Python script. Next, the nnU-Net model inference was performed. Since the nnU-Net model is a volumetric segmentation model, its inference yields a segmentation mask that predicts pulmonary emboli. The segmentation output was transformed into patient-level classification during the post-processing step by applying a threshold to the predicted segmentations, which was based on the total predicted emboli volume. Consequently, a patient-level classification distinguishing between PE and non-PE cases was achieved.

The softmax activation function in the final layer of the U-Net-like architecture provides a probability distribution across predicted classes. By strategically selecting different softmax probability thresholds, it was possible to generate segmentation masks with varying volumes. As such, we established rules that incorporated different softmax probability thresholds (ranging from 0.75 to 0.95 in 0.05 intervals) and volumetric thresholds (ranging from 0 mm^3^ to 200 mm^3^ in 10 mm^3^ intervals). This approach was instrumental in improving the model's performance and fine-tuning the differentiation between PE and non-PE voxel classes. Considering these rules, we formulated two distinct strategies. The strategy that offered the best trade-off between sensitivity and specificity was denoted as Strategy 1, while the one delivering the highest specificity was denoted as Strategy 2 (Supp. materials).

### 2.5 Statistical analysis

Sensitivity and specificity of our trained model for binary classification for PE/non-PE were assessed on a per-patient basis. Matthew's correlation coefficient (MCC) was used to find the optimal balance between sensitivity and specificity. The area under the receiver operating characteristic (AUROC) curve was used to determine classification performance during model training, validation, and evaluation. Statistical analysis was performed with Microsoft Office Excel (Microsoft Corporation, Washington, USA, Office Professional Plus 2016) and statsmodels package (version 0.13.5) in Python (version 3.8.10; Python Software Foundation). A *p*-value less than 0.05 was defined as statistically significant and for C.I., the Wilson score interval was used.

## 3 Results

### 3.1 Model training and performance evaluation on the internal dataset

**Fig. 3:**
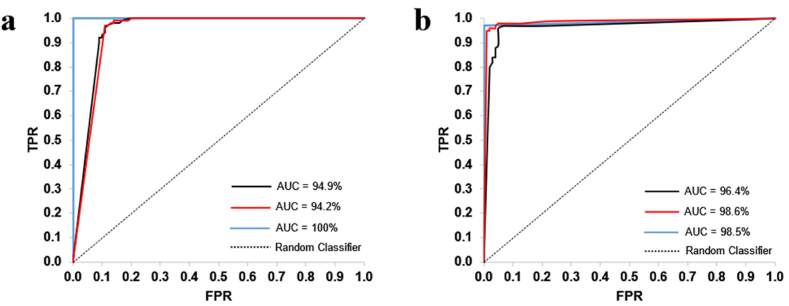
**Classification performance of the trained nnU-Net model.** Area Under the Curves (AUC) without (a) and with (b) post-processing. Black, internal dataset (n = 679, 128 PE and 551 non-PE); Blue, the FUMPE datasets (n = 34, 32 PE and 2 non-PE); Red, the RSNA PE dataset (n = 770, 385 PE and 385 non-PE). TPR, true positive rate; FPR, false positive rate. AUC values are in percentages.

For model training, 2,439,000 voxels of 1497 PE were annotated by two radiologists in all 149 PE positive CTPAs of the internal dataset (Table 1). Acute as well as chronic PEs were annotated, and no distinction was made between them. Consequently, the model did not distinguish between the two types. An nnU-net model was trained with 5-fold cross-validation with 119 training and 30 validation CTPAs per set in 4 sets and 120 training and 29 validation CTPAs in the fifth set without data overlap between the validation sets. To assess model performance, 21 PE positive exams with small PEs (M = 22.9 mm^3^, SD = 11.6 mm^3^) with a total volume of less than 50 mm^3^ were excluded. The remaining 128 PE positive CTPAs and 551 PE negative CTPAs constituted the internal cross-validation and test set. Training and validation was performed on a single Nvidia RTX 2080 TI GPU card which took ∼1 week in total for all cross-validation folds. The classification performance of the trained nnU-Net model on internal and external test datasets was explored over different threshold volumes, with and without post-processing strategies. Without the post-processing strategy and by setting the threshold volume to 20 mm^3^, a Matthews correlation coefficient score (MCC) of 63.9 % was achieved, correctly classifying 128 out of 128 positive examinations as having PE and 433 out of 551 negative examinations as non-PE. With the post-processing strategy 1 (Supp. materials) and threshold volume of 20 mm^3^, the best MCC (84.9 %) was obtained with 123 of 128 positive examinations correctly classified as PE, and 521 of 551 negative examinations correctly classified as non-PE. Further, the model achieved an AUROC of 96.4 % and 94.9 % with and without post-processing respectively (Fig. 3). The trained nnU-Net model thus achieved a sensitivity of 96.1 % (95 % C.I. 91–98 %, *P* < .05) and 100 % (95 % C.I. 97–100 %, *P* < .05), and a specificity of 94.6 % (95 % C.I. 92–96 %, *P* < .05) and 78.6 % (95 % C.I. 75–82 %, *P* < .05) in the internal dataset with and without the post-processing strategies, respectively (Table 2).

**Table 1:** Ground truth annotation of 149 internal CTPAs with PE.

Component	Blood Clots	Average Volume	Min Volume	Max Volume
		(mm^3^)	(mm^3^)	(mm^3^)
3D (Volume)	1497	682	0.21	36510
2D (Area)	36471	16	0.21	830
1D (Voxel)	2439400			

Note. — The total PE volume in all examinations was 744783 mm^3^. PE = Pulmonary embolism, 3D = 3-dimensional, 2D = 2-dimensional, 1D = 1-dimensional. Min = minimum, Max = maximum. 3D components are composed of 2D components, and 2D components are made up of 1D components. A 1D component, in this context, corresponds to a single voxel.

### 3.2 Model performance on external datasets

For external evaluation, the trained model was applied to a total of 804 CTPAs from two publicly available datasets. First, 34 PE positive CTPAs and 2 PE negative CTPAs from the FUMPE dataset were analyzed. With post-processing strategy 1, an MCC score of 80.4 % was obtained with 31 of 32 positive examinations correctly classified as PE, and 2 of 2 negative examinations correctly classified as non-PE. The trained model achieved AUROC 98.5 % (Fig. 3) with sensitivity of 96.9 % (95 % C.I. 84–99 %, *P* < .05) and specificity of 100 % (95 % C.I. 34–100 %, *P* < .05) (Table 2, Supp Table 5). Focusing on central PE, where the annotations can be assumed to be more consistent, we used 385 CTPAs annotated as having at least one central PE and 385 PE negative CTPAs from the RSPECT pulmonary embolism CT dataset for model evaluation. With the post-processing strategy 1 (Supp. Materials), an MCC of 88.6 % was obtained with 379 of 385 positive examinations correctly classified as PE, and 346 of 385 negative examinations correctly classified as non-PE. The trained model achieved an AUROC of 98.6 % (Fig. 3) with sensitivity of 98.4 % (95 % C.I. 97–99 %, *P* < .05) and a specificity of 89.9 % (95 % C.I. 86–93 %, *P* < .05) (Table 2, Supp Table 6). Without the post-processing strategy and by setting the threshold volume to 20 mm^3^, MCC of 100 % and 73.3 % were obtained with 32 (n = 32) and 385 (n = 385) positive examinations correctly classified as PE, and 2 (n = 2) and 269 (n = 385) negative examinations correctly classified as non-PE in the first and second external datasets, respectively (Table 2, Supp Tables 2 and 3). Moreover, the model achieved an AUROC of 100 % and 94.2 % (Fig. 3) with a sensitivity of 100 % (95 % C.I. 89–100 %, *P* < .05) and 100 % (95 % C.I. 99–100 %, *P* < .05), and a specificity of 100 % (95 % C.I. 34–100 %, *P* < .05) and 69.9 % (95 % C.I. 65–74 %, *P* < .05) in the first and second datasets, respectively (Table 2, Supp Tables 2 and 3).

**Table 2:** Diagnostic performance of the trained model.

	Without the Post-Processing			With the Post-Processing		
Parameter	Internal Dataset	FUMPE External Dataset	RSNA External Dataset	Internal Dataset	FUMPE External Dataset	RSNA External Dataset
No. of CTPAs	679	34	770	679	34	770
No. of TN	433	2	269	521	2	346
No. of FP	118	0	116	30	0	39
No. of TP	128	32	385	123	31	379
No. of FN	0	0	0	5	1	6
MCC (%)	63.9 (58–70)	100 (100–100)	73.3 (68–78)	84.9 (81–89)	80.4 (60–100)	88.6 (85–92)
Sensitivity (%)	100 (97–100)	100 (89–100)	100 (99–100)	96.1 (91–98)	96.9 (84–99)	98.4 (97–99)
Specificity (%)	78.6 (75–82)	100 (34–100)	69.9 (65–74)	94.6 (92–96)	100 (34–100)	89.9 (86–93)
Accuracy (%)	82.6 (80–85)	100 (90–100)	84.9 (82–87)	94.8 (92–96)	97.1 (85–99)	94.2 (92–96)
Balanced Accuracy (%)	89.3 (86–91)	100 (62–100)	84.9 (82–87)	95.4 (92–97)	98.4 (59–99)	94.2 (92–95)
AUC (%)	94.9 (77–99)	100 (85–100)	94.2 (76–99)	96.4 (79–99)	98.5 (83–100)	98.6 (83–100)

Note. — The threshold volume is set to 20 mm^3^. Data in parentheses are 95 % CIs in percentages. CTPAs = computed tomography (CT) pulmonary angiography (CTPA) examinations, TN = true-negative CTPAs, FP = false-positive CTPAs, TP = true-positive CTPAs, FN = false-negative CTPAs, MCC = Matthew's correlation coefficient, AUC = area under the receiver operating characteristic curve.

**Fig. 4:**
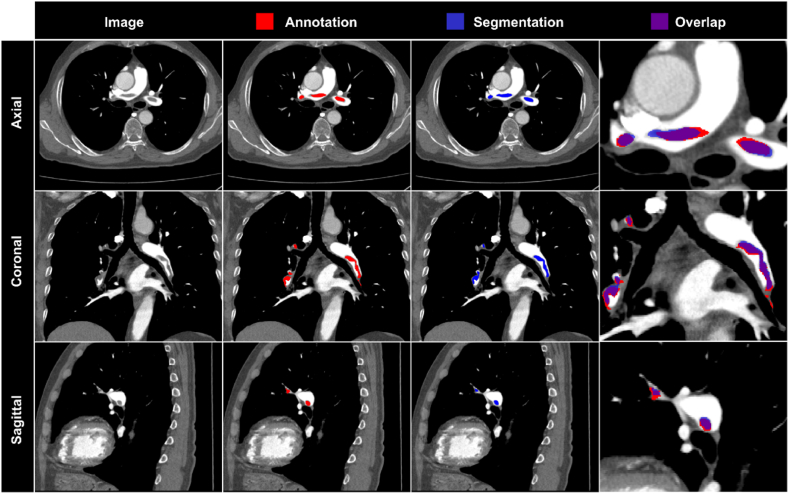
**Representative segmentation results of the trained model.** Axial, coronal, and sagittal planes from the same CTPA examinations from the external FUMPE dataset with the same window setting (width = 800 HU, level = 100 HU) are shown. Red, pulmonary embolism annotation; blue, model segmentation; purple, overlay of annotation and model segmentation.

The output of the automated classification algorithm is shown (Fig. 4, Supp Figures 1-3). Performing model inference within the nnU-Net framework for a single CTPA volume examination, utilizing a singular Nvidia RTX 4090 GPU card, required 240–300 s. Furthermore, disabling the test time augmentation (TTA) yielded a decrease in inference time to 60–70 s with an average accuracy decline of 1 % (Supp Tables 1-12).

### 3.3 Benchmarking of model performance

**Fig. 5:**
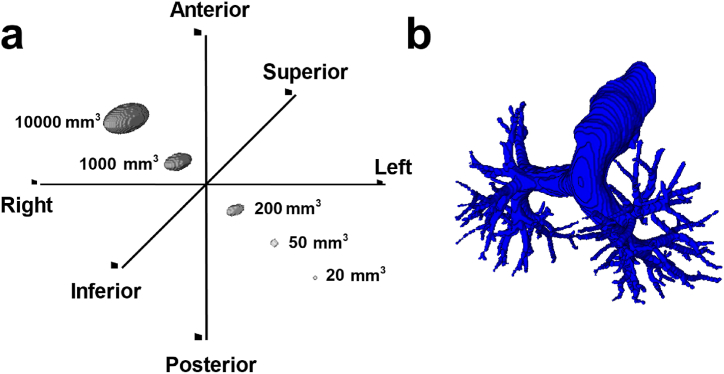
**An illustration of a segmented reference pulmonary artery with reference threshold volumes.** Patient orientation of 3D volumes with reference threshold volumes (20, 50, 200, 1000, and 10000 mm^3^) (a). Manually segmented reference pulmonary artery (volume of 113 cm³) from a male patient without PE (b). All volumetric images are isotropic (1 mm × 1 mm × 1 mm).

As mentioned above, post-processing strategy 1 was used to find out the best balance between sensitivity and specificity and 20 mm^3^ was determined as the optimal threshold volume. Aiming for the highest specificity and the lowest patient level false positive rate, we used post-processing strategy 2 where 50 mm^3^ was determined as optimal threshold volume. For size comparison, 20 mm^3^, 50 mm^3^, and other threshold volumes (Fig. 5a) are compared to a segmented reference pulmonary artery (Fig. 5b). In the internal test dataset, the highest specificity (96.7 %; 95 % C.I. 95–98 %, *P* < .05) was obtained with a sensitivity of 87.5 % (95 % C.I. 81–92 %, *P* < .05) with post-processing strategy 2 (Supp. materials, Supp Table 7) and threshold volume of 50 mm^3^. For the external datasets, the highest specificity 100 % (95 % C.I. 34–100 %, *P* < .05) and 96.9 % (95 % C.I. 95–98 %, *P* < .05) was obtained with a sensitivity of 90.6 % (95 % C.I. 76–97 %, *P* < .05) and 96.6 % (95 % C.I. 94–98 %, *P* < .05) in the FUMPE and RSPECT datasets, respectively (Supp Tables 8 and 9). Moreover, we examined the source of false positives by implementing post-processing strategy 2, which led to a minimum number of FPs per dataset. The most frequent false positives were due to low contrast medium in pulmonary arteries (Table 3). Whereas 18 % of FPs occurred on the outside of the thoracic cavity, in the upper abdomen, or in the superior vena cava, the remaining FPs occurred within or close to the pulmonary vessel network. Besides, most of the false negatives (FN) occurred in the RSPECT dataset, and the primary cause of these FNs is chronic PEs. We next compared model performance to those of previous studies (Table 4). With post-processing strategy 2, the proposed algorithm achieved a sensitivity of 96.2 % (95 % C.I. 94–98 %, *P* < .05) and a specificity of 96.8 % (95 % C.I. 95–98 %, *P* < .05) on the combined (internal and external) testing set (Supp Table 12). While investigating the causes of false positives, we observed that 3 CTPAs from the RSPECT dataset that were annotated as PE negative were actually PE positive. Considering this correction, the proposed algorithm achieved a specificity of 97.1 %.

**Table 3:** Sources of false positives in PE negative CTPA examinations from internal and external datasets.

Source	Internal Dataset (n = 18)	RSPECT External Dataset (n = 12)
Flow artifact	1	1
Upper abdomen (in left colon)	1	0
Outside the thoracic cavity	3	0
Low contrast medium in PT	6	2
Pulmonary vein	1	2
Superior vena cava	1	0
Intrafissural fluid/atelectasis	0	1
Multiple metastasis	1	1
Tumor	4	2
True pulmonary emboli	0	3

Note. — CTPAs = computed tomography (CT) pulmonary angiography (CTPA) examinations, PE = pulmonary embolism, PT = pulmonary trunk, RSPECT = RSNA Pulmonary Embolism CT Dataset. The cause of false positives in a total of 30 CTPAs is shown, 18 in internal and 12 in external datasets.

**Table 4:** Model performance comparison for patient-level classification for PE in CTPA examinations.

								Testing size		
Author	Year	Method	Classification level	PE location	AUC (%)	Sensitivity (%)	Specificity (%)	PE positive CTPAs	PE negative CTPAs	Total # of CTPAs
PIOPED II [29]	2006	Radiologists	patient-level	M, L, S, s	N/A	83	96	181	592	773
Maizlin et al. [30]	2007	IPAT	patient-level	M, L, S, s	N/A	53.3	77.5	15	89	104
Wittenberg et al. [9]	2010	IPAT	patient-level	M, L, S, s	N/A	94	21	68	210	278
Wittenberg et al. [28]	2012	IPAT	patient-level	M, L, S, s	N/A	96	22	51	158	209
Lahiji et al. [31]	2014	IPAT	patient-level	L, S, s	N/A	97.5	26.9	40	26	66
Rajan et al. [16]	2020	2D U-Net + LSTM	patient-level	M, L	85	N/A	N/A	385	127	512
Rajan et al. [16]	2020	2D U-Net + LSTM	patient-level	S, s	70	N/A	N/A	385	127	512
Weikert et al. [17]	2020	DCNN	patient-level	M, L, S, s	N/A	92.7	95.5	232	1233	1465
Weikert et al. [17]	2020	DCNN	patient-level	M, L, (S, s) ∗	N/A	95.7	95.5	232	1233	1465
Weikert et al. [17]	2020	DCNN	patient-level	S, (s)∗	N/A	93.3	95.5	232	1233	1465
Weikert et al. [17]	2020	DCNN	patient-level	s	N/A	85.7	95.5	232	1233	1465
Huang et al. [27]	2020	3D CNN	patient-level	M, L, S	85	75	81	94	106	200
Huhtanen et al. [32]	2022	CNN	patient-level	M, L, S, s	94	86.6	93.5	97	107	204
Ma et al. [33]	2022	TCN + Attention	patient-level	M, L, S, s	91	N/A	N/A	313	687	1000
Wiklund et al. [34]	2023	Commercial	patient-level	M, L, S, s	N/A	90.7	99.8	75	1817	1892
Djahnine et al. [35]	2024	Retina U-Net	patient-level	M, L, S, s	85	N/A	N/A	179^‡^	199^‡^	378
Islam et al. [36]	2024	CNN	patient-level	M, L, S, s	93	N/A	N/A	N/A	N/A	1000
**Proposed pipeline**	**2023**	**nnU-Net + DPPS1**	patient-level	M, L, S, s	**98.2**	**98.3**	**92.6**	**417**	**938**	1355
**Proposed pipeline**	**2023**	**nnU-Net + DPPS2**	patient-level	M, L, S, s	**98.3**	**96.2**	**96.8**	**417**	**938**	1355

Note. — ∗ can possibly have pulmonary emboli in these segments, PE = pulmonary embolism, CTPAs = computed tomography (CT) pulmonary angiography (CTPA) examinations, IPAT = image processing and analysis techniques, N/A = not available, M = left, right and main pulmonary arteries-level PE, L = lobar level PE, S = segmental level PE, s = sub-segmental level PE, LSTM = long short-term memory, CNN = convolutional neural network, DCNN = deep CNN, TCN = temporal convolutional network, PIOPED = prospective investigation of pulmonary embolism diagnosis II, DPPS = deterministic post-processing strategy. ^‡^ A total of 178 CTPAs were conducted, and the numbers of PE and Non-PE exams were estimated from the histogram plot [37].

## 4 Discussion

In this study, we developed an algorithm that classifies CTPA examinations for presence of PE consisting of two main stages, PE candidate selection and post-processing. For PE candidate selection, we trained and validated a semantic segmentation model, nnU-Net, on our internal dataset. The nnU-Net is a medical image segmentation framework based on the U-Net architecture and has outperformed state-of-the-art models by competing in 53 segmentation tasks from 11 international biomedical image segmentation challenges and taking first place in 33 of them [19]. To our knowledge, this is the first use of nnU-Net for classification for PE. To transform the segmentation model into a classification model, we developed rules based on probability and minimum volume thresholds as a post-processing stage. We defined two post-processing strategies, one for the best trade-off between sensitivity and specificity and one for achieving the highest specificity. At the best trade-off between sensitivity and specificity, the patient-level classification performance of the trained model achieved a sensitivity of 98.3 % and specificity of 92.6 % on the combined testing dataset using a threshold volume of 20 mm^3^, compared to specificity of 75.1 % with sensitivity of 100 % without post-processing. Thus, by sacrificing 1.7 % of sensitivity, the model gained 17.4 % in specificity using post-processing.

The methods proposed here show superior performance in pulmonary embolism (PE) detection compared to traditional as well as contemporary approaches. The traditional image processing and analysis techniques methods from Maizlin et al. [30], Wittenberg et al. [9,28], and Lahiji et al. [31] had varied sensitivities (53.3 %–97.5 %) and low specificities (21 %–26.9 %), and were outperformed by the proposed methods. Detection of PE in CTPA using deep convolutional neural networks (DCNN) was first demonstrated by Tajbakhsh et al. with a sensitivity of 83 % and 34.6 % at 2 FPs per examinations on 121 internal and 20 external test examinations, respectively [26]. Rajan et al. proposed a two-stage solution where a 2D U-Net model was used for PE candidate generation, followed by a convolutional long short-term memory (LSTM) network coupled with multiple instance learning to detect PE lesions, with AUROC of 70 % for subsegmental and segmental PE and 0.85 for saddle and main pulmonary artery PE on a test dataset of 512 CTPA examinations [16]. In a study by Huang et al. [27], a 3D CNN model called PENet was developed and tested on an external dataset of 200 CTPA examinations, achieving an AUC of 85 %, a sensitivity of 75 %, and a specificity of 81 %. Other CNN-based methods, such as those from Huhtanen et al., Islam et al., and Ma et al., had AUCs ranging from 91 % to 94 % and thus fall short of the performance demonstrated here. In addition, the studies mentioned above have major limitations such as small test dataset sizes or low specificity rates and the proposed method surpassed all these metrics. The current state-of-the-art results were achieved using Resnet architecture on 1465 CTPA examinations with a sensitivity of 92.7 % and specificity of 95.5 % at the patient level by Weikert et al. [17]. Our model outperformed the current state-of-the-art using the strategy of highest specificity, achieving 96.2 % sensitivity and a specificity of 96.8 % on the combined testing dataset of 1355 CTPA examinations with a total emboli volume threshold of 50 mm^3^. These comparisons highlight the enhanced detection capabilities, accuracy, and reliability of the proposed methods in clinical PE detection. Taken together, the performance of AI systems for PE detection is now at a point where clinical utility can be expected, but further gains in sensitivity and specificity are still warranted.

Treatment for small PEs is debated and controversial [24,25] and PE volumes in this context are rarely measured and reported in the literature and are subject to inter-observer variability. We decided to set a cut-off volume to exclude the very smallest and in some cases doubtful PEs in our internal dataset. Radiologists DT and TF segmented all 1497 PEs and found a total volume of 50 mm^3^ to be a reasonable cut-off for exclusion. In a scenario where the total PE volume of 50 mm^3^ was represented by a single, equidimensional embolus, the cut-off size would amount to a cylinder with a diameter and height of 4 mm. Many of the PEs in the 21 excluded exams were much smaller eccentric or tiny webs, likely chronic.

Although the nnU-Net based model presented here is superior to the state-of-the-art, there are some limitations and opportunities for future enhancement. First, the model was trained on data from a single institution, although derived from five different CT scanners. Second, the training dataset, even though the proportion was not analyzed in detail, was predominantly comprised of acute PEs with a limited representation of chronic PEs. However, our observations suggest that chronic PEs within the RSPECT dataset are a significant factor contributing to false negatives. Third, the RSPECT validation dataset lacks voxel level annotation of PE by radiologists, which precludes final determination of sensitivity and specificity until a review has been completed. Finally, the activation of test time augmentation (TTA) extends the model inference duration to ∼300 s per CTPA examination. Conversely, deactivating the TTA reduces the model inference time to a range of 60–70 s for a single CTPA examination. However, when TTA is disabled, sensitivity and specificity decrease by approximately 0.3 % and 1.7 %, respectively.

In conclusion, nnU-Net deep learning based binary classification for PE holds potential to assist radiologists in the reading of CTPA examinations. Preferentially, such a system could prioritize PE positive cases in the work list, identifying high-priority cases for swift review, or provide a second opinion.

## Data sharing statement

Data generated or analyzed during the study are available from the corresponding author by request.

## Funding information

The project was supported by a grant from Analytic Imaging Diagnostics Arena (AIDA), https://medtech4health.se/aida-en/, to Tobias Sjöblom. Tomas Fröding and Dimitrios Toumpanakis were supported by clinical fellowships from AIDA. Tomas Fröding was supported by the Centre for Clinical Research Sörmland, 10.13039/501100007051Uppsala University, Eskilstuna, Sweden.

## Co-first and Co-senior author contributions

Ali Teymur Kahraman is listed as the first co-first author because of his larger contribution to implementation, computation, and theory development. Tomas Fröding is listed as the second co-first author because of his larger contribution to the data collection and annotation of data sets. Christian Jamtheim Gustafsson and Tobias Sjöblom were listed as co-senior authors due to their equal contribution to the supervision of the project.

## CRediT authorship contribution statement

**Ali Teymur Kahraman:** Writing – review & editing, Writing – original draft, Visualization, Validation, Software, Methodology, Formal analysis, Data curation, Conceptualization. **Tomas Fröding:** Writing – review & editing, Validation, Resources, Investigation, Data curation, Conceptualization. **Dimitris Toumpanakis:** Writing – review & editing, Validation, Data curation. **Christian Jamtheim Gustafsson:** Writing – review & editing, Supervision, Methodology, Conceptualization. **Tobias Sjöblom:** Writing – review & editing, Supervision, Resources, Project administration, Funding acquisition, Conceptualization.

## Declaration of competing interest

The authors declare that they have no conflicts of interest.
