# Supplementary material for: Enhanced classification performance using deep learning based segmentation for pulmonary embolism detection in CT angiography
Source: Heliyon. 2024 Sep 19;10(19):e38118. doi: 10.1016/j.heliyon.2024.e38118 (PMC11471166; doi:10.1016/j.heliyon.2024.e38118)
Supplement: Multimedia component 1 [file mmc1.docx]

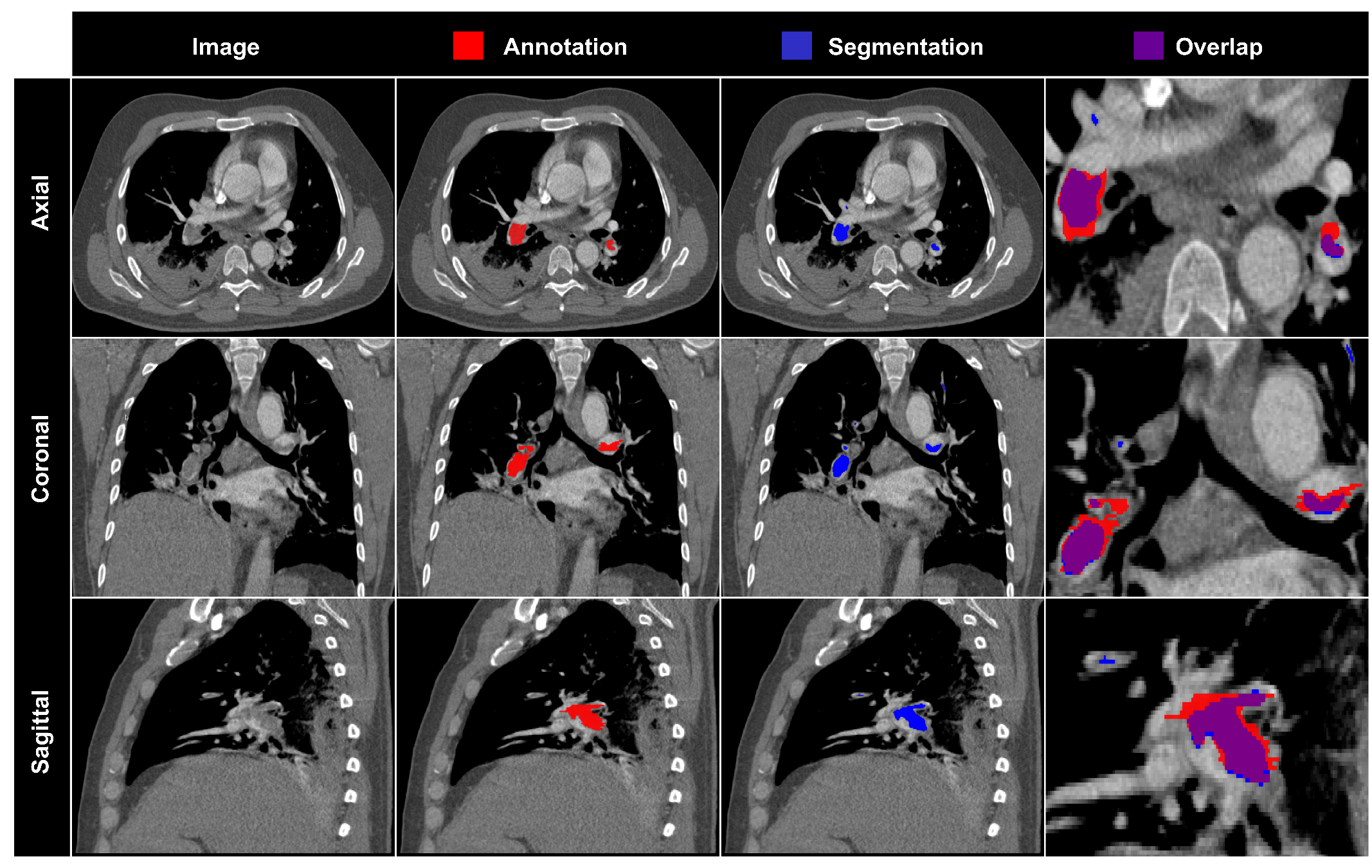


**Supplemental Figure 1. Representative segmentation results from the FUMPE dataset (patient 03).** Axial, coronal, and sagittal planes from the same CTPA examination from the external FUMPE dataset with the same window setting (width = 800 HU, level = 100 HU) are shown. Red, pulmonary embolism annotation; blue, model segmentation; purple, overlay of annotation and model segmentation.


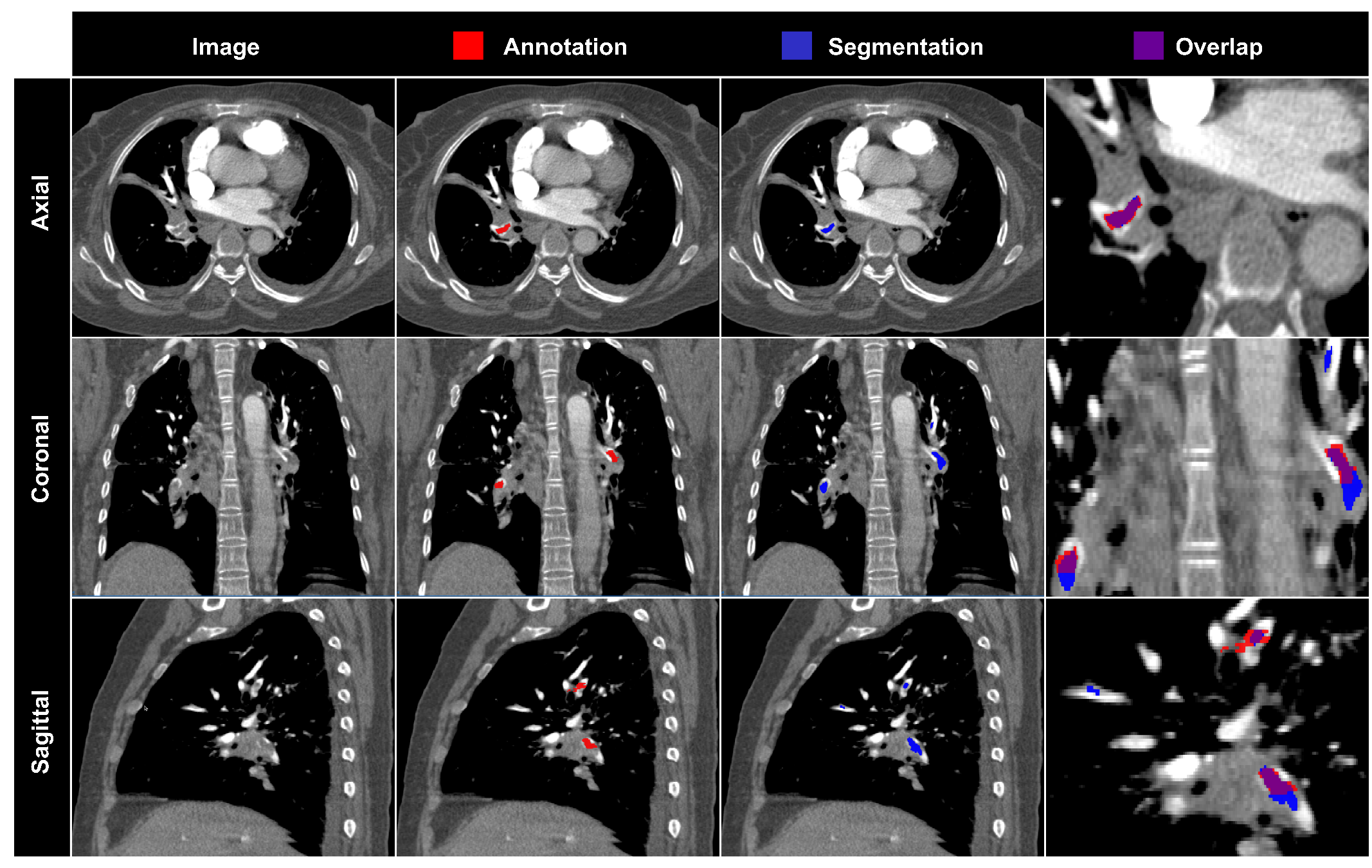


**Supplemental Figure 2. Representative segmentation results from the FUMPE dataset (patient 04).** Axial, coronal, and sagittal planes from the same CTPA examination from the external FUMPE dataset with the same window setting (width = 800 HU, level = 100 HU) are shown. Red, pulmonary embolism annotation; blue, model segmentation; purple, overlay of annotation and model segmentation.


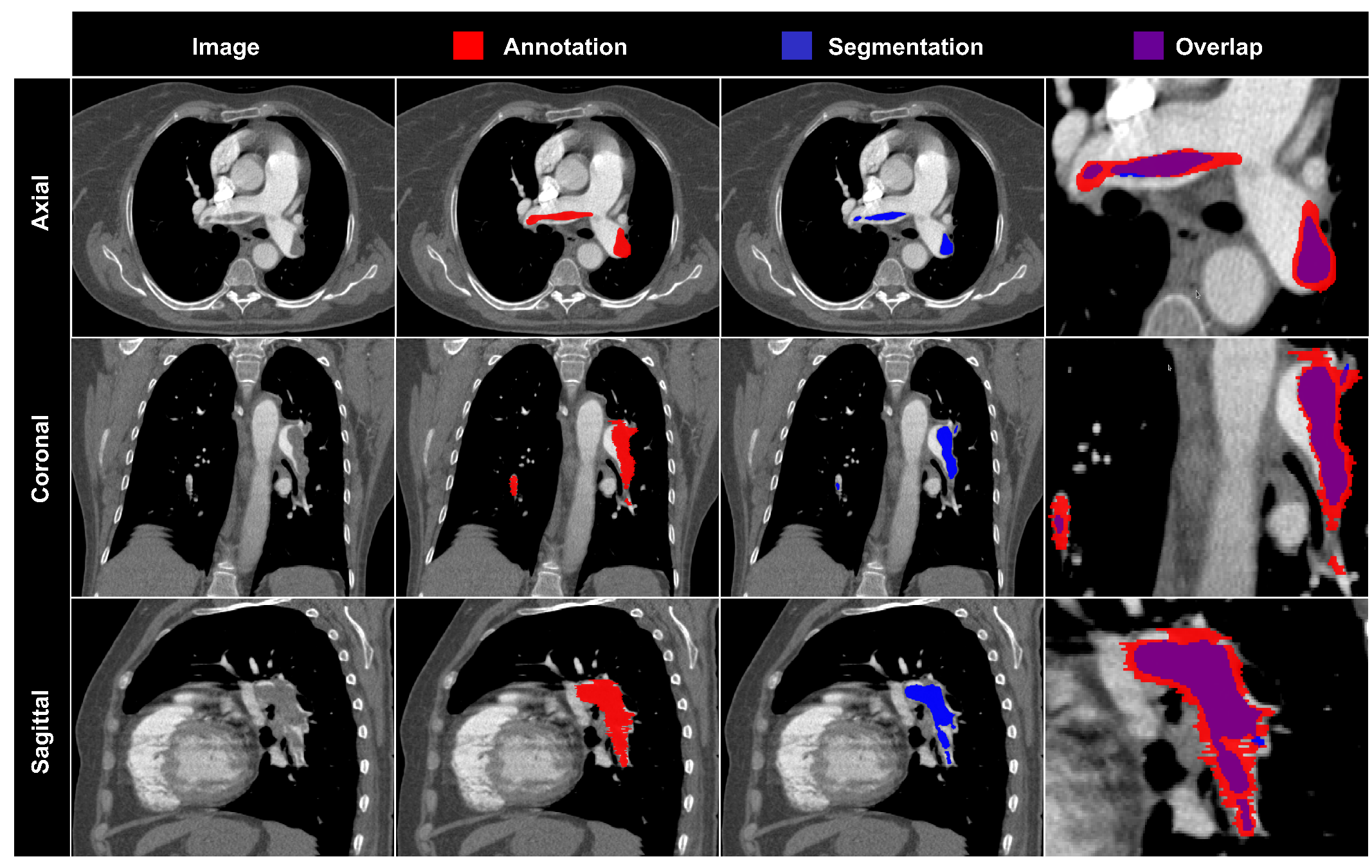


**Supplemental Figure 3. Representative segmentation results from the FUMPE dataset (patient 05).** Axial, coronal, and sagittal planes from the same CTPA examination from the external FUMPE dataset with the same window setting (width = 800 HU, level = 100 HU) are shown. Red, pulmonary embolism annotation; blue, model segmentation; purple, overlay of annotation and model segmentation.


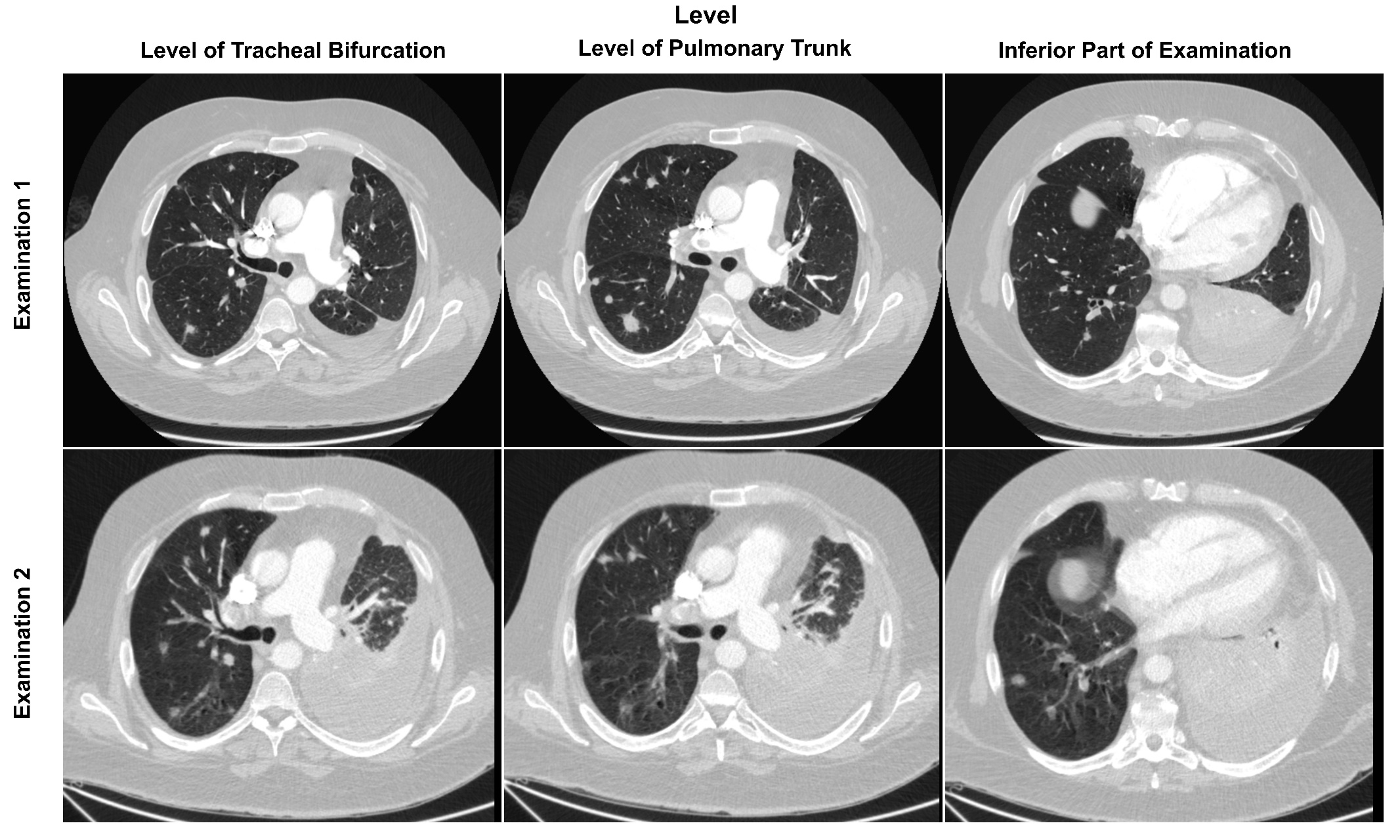


**Supplemental Figure 4. Representative examples from two CT Pulmonary Angiography (CTPA) examinations of the same patient, both having pulmonary embolism.** Two CTPA examinations from the same patient within the internal dataset are presented, featuring identical window settings (width = 1500 HU, level = -400 HU) and depicted at three distinct anatomical levels. The patient exhibited pulmonary embolism in both CTPA examinations.


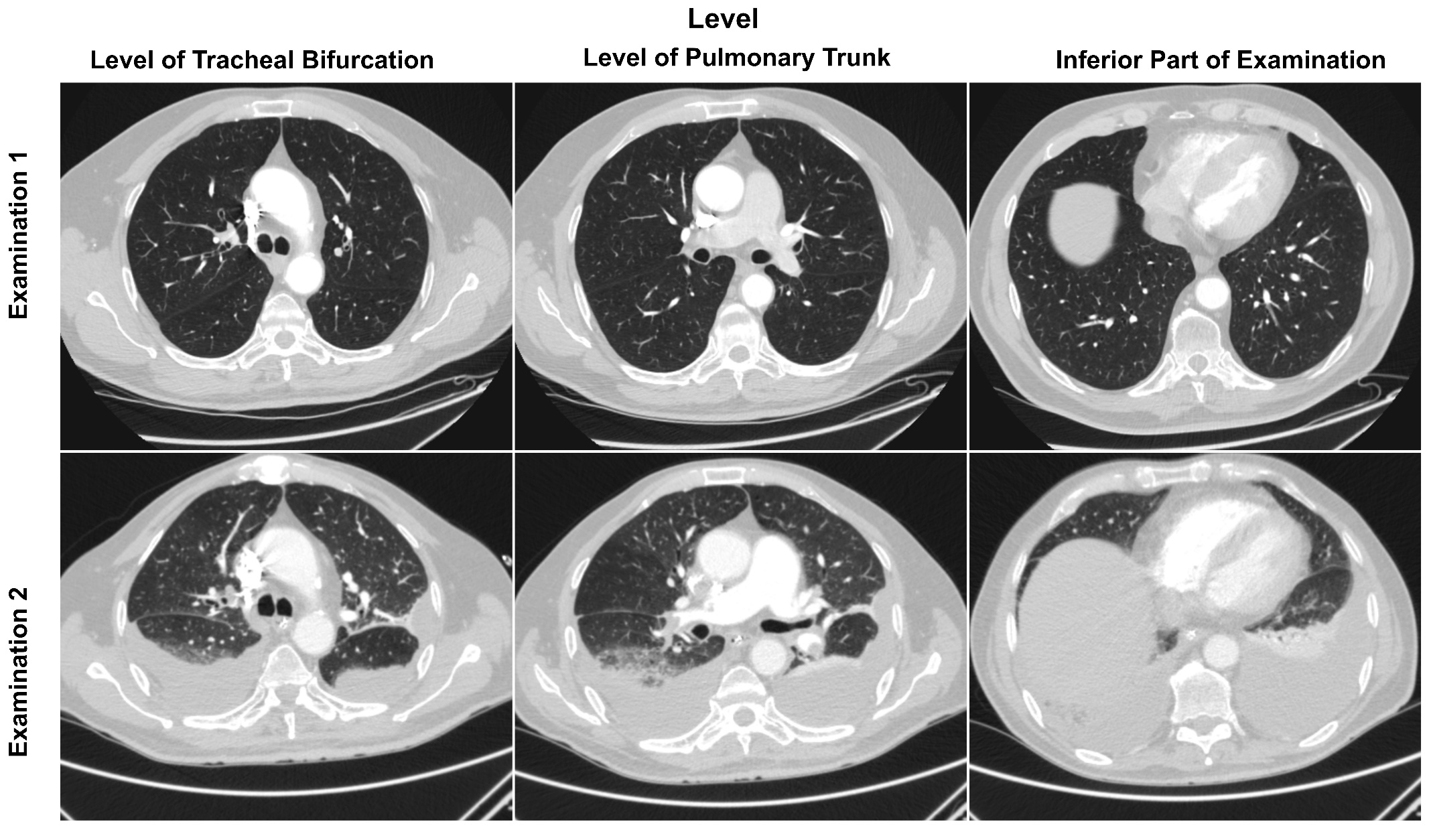


**Supplemental Figure 5. Representative examples of two CT Pulmonary Angiography (CTPA) examinations of the same patient with a pulmonary embolism in one examination but not in the other.** Two CTPA examinations from the same patient within the internal dataset are presented, featuring identical window settings (width = 1500 HU, level = -400 HU) and depicted at three distinct anatomical levels. The patient exhibited pulmonary embolism in examination 2.
