# Supplementary material for: Enhanced classification performance using deep learning based segmentation for pulmonary embolism detection in CT angiography
Source: Heliyon. 2024 Sep 19;10(19):e38118. doi: 10.1016/j.heliyon.2024.e38118 (PMC11471166; doi:10.1016/j.heliyon.2024.e38118)
Supplement: Multimedia component 2 [file mmc2.docx]

**Enhanced Classification Performance using Deep Learning Based Segmentation for Pulmonary Embolism Detection in CT Angiography**

**Supplementary Materials**

**S.1. Dataset**

**S.1.1 Internal dataset CT Acquisition Protocols**

All 700 CTPAs were performed with bolus tracking technique with the region of interest (ROI) in the pulmonary trunk. Different Hounsfield unit thresholds and delays were used. Contrast medium doses were recorded for 191 (range 20 ml – 114 ml, mean 62 ml) and injection rates for 158 (range 2,4 ml/s – 6,1 ml/s, mean 3,6 ml/s) CTPAs, respectively. The most frequently used CT image acquisition parameters were slice thickness 0.625 mm (range 0.625 mm - 2.0 mm), pixel spacing 0.7 mm (range 0.59 mm - 0.98 mm), tube voltage 100 kV (range 80 kV - 120 kV) and scanning direction caudal to cranial. The CTPAs acquired on the Siemens Somatom Definition Flash CT were in the majority of cases performed with dual-energy source acquisition with tube settings 80 kV / 140 kV and the images used in the dataset were post-processed blended images from a weighting factor 0.5.

**S.1.2 Distribution of CT Pulmonary Angiography examinations from the same patient in internal datasets**

The internal dataset comprises 700 CT Pulmonary Angiography (CTPA) examinations involving 652 patients. Among them, 41 patients underwent CTPA twice, 2 patients thrice, and 1 patient four times. Of the 149 pulmonary embolism (PE) -positive examinations, 142 patients were involved, and of the 551 PE-negative examinations, 520 patients were included. Ten patients had both PE-negative and PE-positive CT examinations. Since the CT scans acquired from the same patient were performed at different occasions, several anatomical aspects depending on breath hold level, angle of spine and pulmonary disease status were different (Supp. Figures 4-5). This means that there were differences at the voxel level and also differences in the data label (positive/negative PE), depending on the scan session. The examinations were therefore used and analyzed as if they had been obtained from different patients. They were therefore randomly distributed to cross-validation dataset, regardless of whether they belonged to the same patient. In the external datasets, all CTPAs were obtained from different patients and thus truly statistically independent.

**S.1.3 Ferdowsi University of Mashhad's Pulmonary Embolism Dataset**

The Ferdowsi University of Mashhad's PE dataset (FUMPE) is a publicly available dataset consisting of 35 CTPA examinations with voxel-level PE annotations by radiologists. One PE-positive examination was excluded due to a lack of ground truth annotation. Out of the 34 CTPA examinations, 32 were PE-positive and 2 were PE-negative. When examining the ground truth, we noticed that the slice locations of PE annotations were incorrect in 8 CTPA examinations. Specifically, in these cases, PE annotations that should have been located in slice 101 were mistakenly placed in slice 11. As a result, we relocated the PE annotations from slice 11 to slice 101.

**S.2. Environmental Settings and Versions**

**S.2.1 Model Training Environment**

The environmental settings employed for both model training and cross-validation in this study encompass specific software versions: Ubuntu 22.04.3 LTS as the operating system, Docker version 24.0.7 for containerization, and CUDA Version 12.1, along with Nvidia driver version 530.30.02, are employed to facilitate seamless interactions with the NVIDIA GeForce RTX 2080 Ti GPUs. The programming language employed is Python, specifically version 3.8.10. The deep learning framework PyTorch is leveraged in version 2.0.0, and the semantic segmentation method nnU-Net is implemented in version 1.7.1.

**S.2.2 Model Inference Environment**

For model inference, another workstation was utilized, with specific environmental settings and software versions. These settings include Ubuntu 22.04.3 LTS as the operating system, Docker version 24.0.7 for containerization, CUDA Version 12.2, and Nvidia driver version 535.129.03, facilitating seamless interactions with the NVIDIA GeForce RTX 4090 GPU. The programming language employed is Python version 3.10.6. PyTorch, the deep learning framework, is utilized in version 2.1.0, and the semantic segmentation method nnU-Net is implemented in version 1.7.1.

**S.3. The nnU-Net Deep Learning Framework**

**S.3.1 Hyperparameters**

In the training of our deep learning model, the nnU-Net framework employed a specific set of hyperparameters to optimize the learning process. The chosen optimizer is Stochastic Gradient Descent (SGD) with Nesterov momentum, utilizing a momentum value of 0.99. Additionally, weight decay was incorporated with a coefficient of 3e-05 to regulate the model's complexity during training. The initial learning rate was set to 0.01, providing a starting point for the optimization process. To enhance the training procedure, a learning rate scheduler was implemented with a patience parameter of 30 epochs and a threshold of 1e-06. Lastly, the maximum number of training epochs was defined as 1000. These carefully selected hyperparameters contribute to the fine-tuning of the model, optimizing its performance over the course of training.

**S.3.2 Data Augmentation**

The nnU-Net framework employed a set of data augmentation techniques to generalize the models to prevent overfitting to the training data set. Elastic deformation, a spatial transformation technique, was introduced with an alpha range of (0.0, 200.0) and a sigma range of (9.0, 13.0), implemented with a probability of occurrence set at 0.2. Scaling transformations were applied within the range of (0.7, 1.4). To introduce variability in the orientation of the input data, rotational transformations along the X, Y, and Z axes were implemented with specified ranges. Gamma correction, an intensity transformation, was applied with a probability of 0.3 and a gamma range of (0.7, 1.5). Mirroring along axes (0, 1, 2) were applied. Furthermore, a cascaded random binary transformations and additive brightness adjustments were employed with specified probabilities and parameters.

**S.3.3 3D U-Net Architecture**

The nnU-Net framework is configured to generate a 3D U-Net architecture for semantic segmentation tasks. The 3D U-Net architecture is characterized by a symmetrical design with a decoder path that uses transposed convolutions for up sampling. The decoder path of the network consists of five transposed convolutional layers. Each layer employs 3D transposed convolutional operation with varying input and output channel sizes, effectively increasing spatial resolution. Starting with the first layer, it utilizes a transposed convolution operation with 320 input channels, 320 output channels, a 2x2x2 kernel size, and a stride of 2 in all spatial dimensions. Subsequent other layers follow a similar structure, progressively decreasing the number of input channels while maintaining the up-sampling strategy. Additionally, the architecture includes an encoder path with five convolutional layers, where each 3D convolutional operation employs a 1x1x1 kernel with a stride of 1. These layers reduce the channel depth and capture hierarchical features. Each convolutional layer followed by 3D instance normalization and leaky rectified linear unit activation.

**S.4. Post-processing step**

The nn-Unet *softmax* activation function of the final layer of the U-Net architecture can be used to scale network output into probabilities. Hence, the probabilities could be gathered, and not only final pixel class values. We developed a set of logical rules based on different *softmax* probability thresholds (0.75 - 0.95) and threshold volumes per examinations (0 mm³ to 200 mm³ in 10 mm³ intervals) to reduce false positives (FPs) and convert nnU-Net inference segmentation output into a patient-level classification output. By setting different *softmax* probability thresholds, we obtained different predicted PE volumes. If the model is well-trained to distinguish between PE and non-PE classes, the number of predicted voxels (false positive voxels) that do not belong to the PE class will decrease when the *softmax* probabilities are set to higher thresholds. Therefore, we developed the formulas below to decide whether the total predicted PE volume is sufficient to determine the patient as PE positive/negative.

Proposition 1:

$$R= \left\{ \begin{aligned} \\ &if \frac{\left( P_{0.75}- P_{0.90} \right)}{P_{0.90}}> r , Non-PE \\ otherwise , PE \\ \end{aligned} \right.$$

where $P_{0.75}$ is the volume of total PE predicted by the trained model at a softmax probability of 0.75, $P_{0.90}$ is the volume of total PE predicted by the trained model at a *softmax* probability of 0.90, and $r$ is the ratio factor, which was fixed at 15. The *softmax* probability value range and the ratio factor were optimized by systematic exploration.

Proposition 2:

$$Q= \left\{ \begin{aligned} \\ \left( \sum_{i=0.75}^{0.95} \left( \begin{matrix} 1, & if \left( P_{i}<v \right) \\ 0, & otherwise \end{matrix} \right) \right) \geq k , Non-PE \\ otherwise , PE \\ \end{aligned} \right.$$

where $P_{i}$ is the volume of total PE predicted by the trained model at a *softmax* probability of *i* between 0.75 to 0.95 with 0.05 intervals, *v* is the threshold volume between 0 and 200 mm³ at 10 mm³ intervals and $k$ is the condition factor (min value is 0, max value is 4) that refers to the total number of true conditions satisfying $P_{i}<v$ equation.

Then, the final decision is made as follows:

$$R \vee Q= \left\{ \begin{aligned} \\ Patient without PE, True \\ Patient with PE, False \\ \end{aligned} \right.$$

According to the propositions above, we defined two post-processing strategies. Strategy 1 (Rule-in classification for PE) aimed to find the exact threshold volume value and $k$ value for the best trade-off between sensitivity and specificity by checking the Matthew’s correlation coefficient (MCC) value. And strategy 2 (Rule-out classification for PE) aimed to find the exact threshold volume and $k$ values for the highest specificity alongside the highest MCC value.

*Strategy 1:*

By systematic exploration, setting the threshold volume value to 20 mm³ and the k value to 1 gives the highest MCC value (84.9%, Supplementary Table 4).

*Strategy 2*

By systematic exploration, setting the threshold volume value to 50 mm³ and the k value to 0 gives the highest specificity alongside the highest MCC value (83.7%, Supplementary Table 7).
