# Supplementary material for: Enhanced classification performance using deep learning based segmentation for pulmonary embolism detection in CT angiography
Source: Heliyon. 2024 Sep 19;10(19):e38118. doi: 10.1016/j.heliyon.2024.e38118 (PMC11471166; doi:10.1016/j.heliyon.2024.e38118)
Supplement: Multimedia component 3 [file mmc3.docx]

***Supplementary Table 1. Diagnostic performance of the trained model without post-processing in the internal dataset***

|  | Internal Dataset (CTPAs = 679) | | | | | | | | | | | | | | | | | | | | |  |
| --- | --- | --- | --- | --- | --- | --- | --- | --- | --- | --- | --- | --- | --- | --- | --- | --- | --- | --- | --- | --- | --- | --- |
|  | Test Time Augmentation Enabled | | | | | | | | | | | | | | | | | | | | |  |
| Metric \ Threshold | 0 | 10 | 20 | 30 | 40 | 50 | 60 | 70 | 80 | 90 | 100 | 110 | 120 | 130 | 140 | 150 | 160 | 170 | 180 | 190 | 200 |  |
| No. of TN | 213 | 389 | 433 | 459 | 465 | 476 | 486 | 488 | 490 | 492 | 493 | 496 | 497 | 499 | 501 | 501 | 502 | 502 | 502 | 503 | 503 |  |
| No. of FP | 338 | 162 | 118 | 92 | 86 | 75 | 65 | 63 | 61 | 59 | 58 | 55 | 54 | 52 | 50 | 50 | 49 | 49 | 49 | 48 | 48 |  |
| No. of TP | 128 | 128 | 128 | 127 | 126 | 126 | 124 | 123 | 123 | 121 | 120 | 119 | 118 | 118 | 118 | 117 | 117 | 116 | 116 | 116 | 115 |  |
| No. of FN | 0 | 0 | 0 | 1 | 2 | 2 | 4 | 5 | 5 | 7 | 8 | 9 | 10 | 10 | 10 | 11 | 11 | 12 | 12 | 12 | 13 |  |
| MCC (%) | 32.6 | 55.8 | 63.9 | 69.0 | 69.9 | 72.7 | 74.2 | 74.2 | 74.8 | 74.3 | 74.0 | 74.3 | 74.1 | 74.7 | 75.3 | 74.8 | 75.1 | 74.5 | 74.5 | 74.9 | 74.3 |  |
| Sensitivity (%) | 100 | 100 | 100 | 99 | 98.4 | 98.4 | 96.9 | 96.1 | 96.1 | 94.5 | 93.8 | 93.0 | 92.2 | 92.2 | 92.2 | 91.4 | 91.4 | 90.6 | 90.6 | 90.6 | 89.8 |  |
| Specificity (%) | 38.7 | 70.6 | 78.6 | 83.3 | 84.4 | 86.4 | 88.2 | 88.6 | 88.9 | 89.3 | 89.5 | 90.0 | 90.2 | 90.6 | 90.9 | 90.9 | 91.1 | 91.1 | 91.1 | 91.3 | 91.3 |  |
| Accuracy (%) | 50.2 | 76.1 | 82.6 | 86.3 | 87.0 | 88.7 | 89.8 | 90.0 | 90.3 | 90.3 | 90.3 | 90.6 | 90.6 | 90.9 | 91.2 | 91.0 | 91.2 | 91.0 | 91.0 | 91.2 | 91.0 |  |
| Balanced Accuracy (%) | 69.4 | 85.3 | 89.3 | 91.2 | 91.4 | 92.4 | 92.6 | 92.4 | 92.5 | 91.9 | 91.6 | 91.5 | 91.2 | 91.4 | 91.6 | 91.2 | 91.2 | 90.9 | 90.9 | 91.0 | 90.6 |  |
|  | Test Time Augmentation Disabled | | | | | | | | | | | | | | | | | | | | |  |
| No. of TN | 189 | 378 | 417 | 440 | 450 | 462 | 473 | 478 | 480 | 487 | 490 | 490 | 492 | 492 | 493 | 494 | 496 | 499 | 500 | 502 | 502 |  |
| No. of FP | 362 | 173 | 134 | 111 | 101 | 89 | 78 | 73 | 71 | 64 | 61 | 61 | 59 | 59 | 58 | 57 | 55 | 52 | 51 | 49 | 49 |  |
| No. of TP | 128 | 128 | 128 | 128 | 127 | 126 | 125 | 123 | 122 | 121 | 119 | 119 | 118 | 118 | 118 | 118 | 117 | 116 | 116 | 115 | 115 |  |
| No. of FN | 0 | 0 | 0 | 0 | 1 | 2 | 3 | 5 | 6 | 7 | 9 | 9 | 10 | 10 | 10 | 10 | 11 | 12 | 12 | 13 | 13 |  |
| MCC (%) | 29.9 | 54.0 | 60.8 | 65.4 | 67.0 | 69.2 | 71.3 | 71.5 | 71.5 | 72.8 | 72.6 | 72.6 | 72.6 | 72.6 | 72.9 | 73.2 | 73.2 | 73.6 | 73.9 | 74.0 | 74.0 |  |
| Sensitivity (%) | 100 | 100 | 100 | 100 | 99.2 | 98.4 | 97.7 | 96.1 | 95.3 | 94.5 | 93.0 | 93.0 | 92.2 | 92.2 | 92.2 | 92.2 | 91.4 | 90.6 | 90.6 | 89.8 | 89.8 |  |
| Specificity (%) | 34.3 | 68.6 | 75.7 | 79.9 | 81.7 | 83.8 | 85.8 | 86.8 | 87.1 | 88.4 | 88.9 | 88.9 | 89.3 | 89.3 | 89.5 | 89.7 | 90.0 | 90.6 | 90.7 | 91.1 | 91.1 |  |
| Accuracy (%) | 46.7 | 74.5 | 80.3 | 83.7 | 85.0 | 86.6 | 88.1 | 88.5 | 88.7 | 89.5 | 89.7 | 89.7 | 89.8 | 89.8 | 90.0 | 90.1 | 90.3 | 90.6 | 90.7 | 90.9 | 90.9 |  |
| Balanced Accuracy (%) | 67.2 | 84.3 | 87.8 | 90.0 | 90.4 | 91.1 | 91.8 | 91.4 | 91.2 | 91.4 | 91.0 | 91.0 | 90.8 | 90.8 | 90.8 | 91.0 | 90.7 | 90.6 | 90.6 | 90.4 | 90.4 |  |
| Note. — The thresholds are in mm³. CTPAs = computed tomography (CT) pulmonary angiography (CTPA) examinations, TN = true-negative CTPAs, FP = false-positive CTPAs,  TP = true-positive CTPAs, FN = false-negative CTPAs, MCC = Matthew’s correlation coefficient. | | | | | | | | | | | | | | | | | | | | | |  |
|  |  |  |  |  |  |  |  |  |  |  |  |  |  |  |  |  |  |  |  |  |  |  |
|  |  |  |  |  |  |  |  |  |  |  |  |  |  |  |  |  |  |  |  |  |  |  |

***Supplementary Table 2. Diagnostic performance of the trained model without post-processing in the external FUMPE dataset***

|  | FUMPE External Dataset (CTPAs = 34) | | | | | | | | | | | | | | | | | | | | |  |
| --- | --- | --- | --- | --- | --- | --- | --- | --- | --- | --- | --- | --- | --- | --- | --- | --- | --- | --- | --- | --- | --- | --- |
|  | Test Time Augmentation Enabled | | | | | | | | | | | | | | | | | | | | |  |
| Metric \ Threshold | 0 | 10 | 20 | 30 | 40 | 50 | 60 | 70 | 80 | 90 | 100 | 110 | 120 | 130 | 140 | 150 | 160 | 170 | 180 | 190 | 200 |  |
| No. of TN | 2 | 2 | 2 | 2 | 2 | 2 | 2 | 2 | 2 | 2 | 2 | 2 | 2 | 2 | 2 | 2 | 2 | 2 | 2 | 2 | 2 |  |
| No. of FP | 0 | 0 | 0 | 0 | 0 | 0 | 0 | 0 | 0 | 0 | 0 | 0 | 0 | 0 | 0 | 0 | 0 | 0 | 0 | 0 | 0 |  |
| No. of TP | 32 | 32 | 32 | 32 | 32 | 32 | 32 | 32 | 32 | 32 | 31 | 31 | 30 | 30 | 29 | 29 | 29 | 29 | 29 | 29 | 29 |  |
| No. of FN | 0 | 0 | 0 | 0 | 0 | 0 | 0 | 0 | 0 | 0 | 1 | 1 | 2 | 2 | 3 | 3 | 3 | 3 | 3 | 3 | 3 |  |
| MCC (%) | 100.0 | 100 | 100 | 100 | 100 | 100 | 100 | 100 | 100 | 100 | 80 | 80 | 69 | 69 | 60 | 60.2 | 60.2 | 60.2 | 60.2 | 60.2 | 60.2 |  |
| Sensitivity (%) | 100 | 100 | 100 | 100 | 100 | 100 | 100 | 100 | 100 | 100 | 97 | 97 | 94 | 94 | 91 | 90.6 | 90.6 | 90.6 | 90.6 | 90.6 | 90.6 |  |
| Specificity (%) | 100 | 100 | 100 | 100 | 100 | 100 | 100 | 100 | 100 | 100 | 100 | 100 | 100 | 100 | 100 | 100 | 100 | 100 | 100 | 100 | 100 |  |
| Accuracy (%) | 100 | 100 | 100 | 100 | 100 | 100 | 100 | 100 | 100 | 100 | 97 | 97 | 94 | 94 | 91 | 91.2 | 91.2 | 91.2 | 91.2 | 91.2 | 91.2 |  |
| Balanced Accuracy (%) | 100 | 100 | 100 | 100 | 100 | 100 | 100 | 100 | 100 | 100 | 98 | 98 | 97 | 97 | 95 | 95.3 | 95.3 | 95.3 | 95.3 | 95.3 | 95.3 |  |
|  | Test Time Augmentation Disabled | | | | | | | | | | | | | | | | | | | | |  |
| No. of TN | 1 | 2 | 2 | 2 | 2 | 2 | 2 | 2 | 2 | 2 | 2 | 2 | 2 | 2 | 2 | 2 | 2 | 2 | 2 | 2 | 2 |  |
| No. of FP | 1 | 0 | 0 | 0 | 0 | 0 | 0 | 0 | 0 | 0 | 0 | 0 | 0 | 0 | 0 | 0 | 0 | 0 | 0 | 0 | 0 |  |
| No. of TP | 32 | 32 | 32 | 32 | 32 | 32 | 32 | 32 | 32 | 32 | 32 | 32 | 32 | 32 | 32 | 30 | 30 | 30 | 30 | 30 | 30 |  |
| No. of FN | 0 | 0 | 0 | 0 | 0 | 0 | 0 | 0 | 0 | 0 | 0 | 0 | 0 | 0 | 0 | 2 | 2 | 2 | 2 | 2 | 2 |  |
| MCC (%) | 69.6 | 100 | 100 | 100 | 100 | 100 | 100 | 100 | 100 | 100 | 100 | 100 | 100 | 100 | 100 | 68.5 | 68.5 | 68.5 | 68.5 | 68.5 | 68.5 |  |
| Sensitivity (%) | 100 | 100 | 100 | 100 | 100 | 100 | 100 | 100 | 100 | 100 | 100 | 100 | 100 | 100 | 100 | 93.8 | 93.8 | 93.8 | 93.8 | 93.8 | 93.8 |  |
| Specificity (%) | 50.0 | 100 | 100 | 100 | 100 | 100 | 100 | 100 | 100 | 100 | 100 | 100 | 100 | 100 | 100 | 100 | 100 | 100 | 100 | 100 | 100 |  |
| Accuracy (%) | 97.1 | 100 | 100 | 100 | 100 | 100 | 100 | 100 | 100 | 100 | 100 | 100 | 100 | 100 | 100 | 94.1 | 94.1 | 94.1 | 94.1 | 94.1 | 94.1 |  |
| Balanced Accuracy (%) | 75.0 | 100 | 100 | 100 | 100 | 100 | 100 | 100 | 100 | 100 | 100 | 100 | 100 | 100 | 100 | 96.9 | 96.9 | 96.9 | 96.9 | 96.9 | 96.9 |  |
| Note. — The thresholds are in mm³. CTPAs = computed tomography (CT) pulmonary angiography (CTPA) examinations, TN = true-negative CTPAs, FP = false-positive CTPAs, TP = true-positive CTPAs, FN = false-negative CTPAs, MCC = Matthew’s correlation coefficient. FUMPE = Ferdowsi University of Mashhad's PE dataset. | | | | | | | | | | | | | | | | | | | | | |  |
|  |  |  |  |  |  |  |  |  |  |  |  |  |  |  |  |  |  |  |  |  |  |  |
|  |  |  |  |  |  |  |  |  |  |  |  |  |  |  |  |  |  |  |  |  |  |  |

***Supplementary Table 3. Diagnostic performance of the trained model without post-processing in the external RSPECT Dataset***

|  | RSPECT External Dataset (CTPAs = 770) | | | | | | | | | | | | | | | | | | | | |  |
| --- | --- | --- | --- | --- | --- | --- | --- | --- | --- | --- | --- | --- | --- | --- | --- | --- | --- | --- | --- | --- | --- | --- |
|  | Test Time Augmentation Enabled | | | | | | | | | | | | | | | | | | | | |  |
| Metric \ Threshold | 0 | 10 | 20 | 30 | 40 | 50 | 60 | 70 | 80 | 90 | 100 | 110 | 120 | 130 | 140 | 150 | 160 | 170 | 180 | 190 | 200 |  |
| No. of TN | 131 | 249 | 269 | 287 | 297 | 303 | 308 | 312 | 314 | 318 | 321 | 326 | 328 | 330 | 333 | 333 | 335 | 337 | 338 | 341 | 343 |  |
| No. of FP | 254 | 136 | 116 | 98 | 88 | 82 | 77 | 73 | 71 | 67 | 64 | 59 | 57 | 55 | 52 | 52 | 50 | 48 | 47 | 44 | 42 |  |
| No. of TP | 385 | 385 | 385 | 385 | 385 | 385 | 384 | 383 | 382 | 382 | 382 | 382 | 382 | 380 | 376 | 376 | 376 | 375 | 374 | 374 | 374 |  |
| No. of FN | 0 | 0 | 0 | 0 | 0 | 0 | 1 | 2 | 3 | 3 | 3 | 3 | 3 | 5 | 9 | 9 | 9 | 10 | 11 | 11 | 11 |  |
| MCC (%) | 45.3 | 69.1 | 73.3 | 77.1 | 79.2 | 80.5 | 81.3 | 81.9 | 82.1 | 83.0 | 83.7 | 84.8 | 85.3 | 85.1 | 84.7 | 84.7 | 85.2 | 85.4 | 85.3 | 86.0 | 86.5 |  |
| Sensitivity (%) | 100 | 100 | 100 | 100 | 100 | 100 | 99.7 | 99.5 | 99.2 | 99.2 | 99.2 | 99.2 | 99.2 | 98.7 | 97.7 | 97.7 | 97.7 | 97.4 | 97.1 | 97.1 | 97.1 |  |
| Specificity (%) | 34.0 | 64.7 | 69.9 | 74.5 | 77.1 | 78.7 | 80.0 | 81.0 | 81.6 | 82.6 | 83.4 | 84.7 | 85.2 | 85.7 | 86.5 | 86.5 | 87.0 | 87.5 | 87.8 | 88.6 | 89.1 |  |
| Accuracy (%) | 67.0 | 82.3 | 84.9 | 87.3 | 88.6 | 89.4 | 89.9 | 90.3 | 90.4 | 90.9 | 91.3 | 91.9 | 92.2 | 92.2 | 92.1 | 92.1 | 92.3 | 92.5 | 92.5 | 92.9 | 93.1 |  |
| Balanced Accuracy (%) | 67.0 | 82.4 | 84.9 | 87.2 | 88.6 | 89.4 | 89.8 | 90.2 | 90.4 | 90.9 | 91.3 | 92.0 | 92.2 | 92.2 | 92.1 | 92.1 | 92.4 | 92.4 | 92.4 | 92.8 | 93.1 |  |
|  | Test Time Augmentation Disabled | | | | | | | | | | | | | | | | | | | | |  |
| No. of TN | 118 | 235 | 261 | 276 | 288 | 293 | 304 | 306 | 312 | 315 | 317 | 320 | 325 | 330 | 332 | 333 | 334 | 337 | 338 | 339 | 339 |  |
| No. of FP | 267 | 150 | 124 | 109 | 97 | 92 | 81 | 79 | 73 | 70 | 68 | 65 | 60 | 55 | 53 | 52 | 51 | 48 | 47 | 46 | 46 |  |
| No. of TP | 385 | 385 | 385 | 385 | 385 | 384 | 384 | 383 | 383 | 383 | 383 | 383 | 382 | 382 | 379 | 379 | 377 | 376 | 376 | 376 | 376 |  |
| No. of FN | 0 | 0 | 0 | 0 | 0 | 1 | 1 | 2 | 2 | 2 | 2 | 2 | 3 | 3 | 6 | 6 | 8 | 9 | 9 | 9 | 9 |  |
| MCC (%) | 42.5 | 66 | 72 | 75 | 77 | 78 | 81 | 81 | 82 | 83 | 83 | 84 | 85 | 86 | 85 | 85.5 | 85.2 | 85.6 | 85.9 | 86.1 | 86.1 |  |
| Sensitivity (%) | 100 | 100 | 100 | 100 | 100 | 100 | 100 | 100 | 100 | 100 | 100 | 100 | 99 | 99 | 98 | 98.4 | 97.9 | 97.7 | 97.7 | 97.7 | 97.7 |  |
| Specificity (%) | 30.6 | 61 | 68 | 72 | 75 | 76 | 79 | 80 | 81 | 82 | 82 | 83 | 84 | 86 | 86 | 87 | 87 | 88 | 88 | 88 | 88 |  |
| Accuracy (%) | 65.3 | 81 | 84 | 86 | 87 | 88 | 89 | 90 | 90 | 91 | 91 | 91 | 92 | 93 | 92 | 92.5 | 92.3 | 92.6 | 92.7 | 92.9 | 92.9 |  |
| Balanced Accuracy (%) | 65.3 | 81 | 84 | 86 | 87 | 88 | 89 | 90 | 90 | 91 | 91 | 91 | 92 | 92 | 92 | 92.4 | 92.4 | 92.6 | 92.8 | 92.9 | 92.9 |  |
| Note. — The thresholds are in mm³. CTPAs = computed tomography (CT) pulmonary angiography (CTPA) examinations, TN = true-negative CTPAs, FP = false-positive CTPAs, TP = true-positive CTPAs, FN = false-negative CTPAs, MCC = Matthew’s correlation coefficient. RSPECT = RSNA Pulmonary Embolism CT Dataset. | | | | | | | | | | | | | | | | | | | | | |  |
|  |  |  |  |  |  |  |  |  |  |  |  |  |  |  |  |  |  |  |  |  |  |  |
|  |  |  |  |  |  |  |  |  |  |  |  |  |  |  |  |  |  |  |  |  |  |  |

***Supplementary Table 4. Diagnostic performance of the trained model with post-processing strategy 1 in the internal dataset***

|  | Internal Dataset (CTPAs = 679) | | | | | | | | | | | | | | | | | | | | |  |
| --- | --- | --- | --- | --- | --- | --- | --- | --- | --- | --- | --- | --- | --- | --- | --- | --- | --- | --- | --- | --- | --- | --- |
|  | Test Time Augmentation Enabled | | | | | | | | | | | | | | | | | | | | |  |
| Metric \ Threshold | 0 | 10 | 20 | 30 | 40 | 50 | 60 | 70 | 80 | 90 | 100 | 110 | 120 | 130 | 140 | 150 | 160 | 170 | 180 | 190 | 200 |  |
| No. of TN | 439 | 511 | 521 | 523 | 526 | 529 | 529 | 530 | 530 | 530 | 531 | 533 | 534 | 535 | 536 | 536 | 537 | 538 | 538 | 538 | 538 |  |
| No. of FP | 112 | 40 | 30 | 28 | 25 | 22 | 22 | 21 | 21 | 21 | 20 | 18 | 17 | 16 | 15 | 15 | 14 | 13 | 13 | 13 | 13 |  |
| No. of TP | 124 | 124 | 123 | 120 | 114 | 113 | 113 | 111 | 109 | 108 | 108 | 108 | 106 | 106 | 106 | 105 | 105 | 102 | 102 | 102 | 101 |  |
| No. of FN | 4 | 4 | 5 | 8 | 14 | 15 | 15 | 17 | 19 | 20 | 20 | 20 | 22 | 22 | 22 | 23 | 23 | 26 | 26 | 26 | 27 |  |
| MCC (%) | 62.9 | 81.9 | 84.9 | 84.0 | 81.9 | 82.6 | 82.6 | 81.9 | 80.9 | 80.3 | 80.7 | 81.6 | 81.0 | 81.4 | 81.9 | 81.3 | 81.8 | 80.6 | 80.6 | 80.6 | 80.1 |  |
| Sensitivity (%) | 96.9 | 96.9 | 96.1 | 93.8 | 89.1 | 88.3 | 88.3 | 86.7 | 85.2 | 84.4 | 84.4 | 84.4 | 82.8 | 82.8 | 82.8 | 82.0 | 82.0 | 79.7 | 79.7 | 79.7 | 78.9 |  |
| Specificity (%) | 79.7 | 92.7 | 94.6 | 94.9 | 95.5 | 96.0 | 96.0 | 96.2 | 96.2 | 96.2 | 96.4 | 96.7 | 96.9 | 97.1 | 97.3 | 97.3 | 97.5 | 97.6 | 97.6 | 97.6 | 97.6 |  |
| Accuracy (%) | 82.9 | 93.5 | 94.8 | 94.7 | 94.3 | 94.6 | 94.6 | 94.4 | 94.1 | 94.0 | 94.1 | 94.4 | 94.3 | 94.4 | 94.6 | 94.4 | 94.6 | 94.3 | 94.3 | 94.3 | 94.1 |  |
| Balanced Accuracy (%) | 88.3 | 94.8 | 95.4 | 94.4 | 92.3 | 92.2 | 92.2 | 91.4 | 90.7 | 90.3 | 90.4 | 90.6 | 89.8 | 89.9 | 90.0 | 89.6 | 89.8 | 88.6 | 88.6 | 88.6 | 88.2 |  |
|  | Test Time Augmentation Disabled | | | | | | | | | | | | | | | | | | | | |  |
| No. of TN | 402 | 502 | 512 | 515 | 519 | 524 | 525 | 526 | 526 | 527 | 529 | 529 | 530 | 531 | 532 | 532 | 532 | 534 | 535 | 535 | 535 |  |
| No. of FP | 149 | 49 | 39 | 36 | 32 | 27 | 26 | 25 | 25 | 24 | 22 | 22 | 21 | 20 | 19 | 19 | 19 | 17 | 16 | 16 | 16 |  |
| No. of TP | 125 | 125 | 124 | 121 | 117 | 115 | 115 | 113 | 111 | 110 | 110 | 108 | 106 | 106 | 106 | 105 | 105 | 103 | 103 | 102 | 102 |  |
| No. of FN | 3 | 3 | 4 | 7 | 11 | 13 | 13 | 15 | 17 | 18 | 18 | 20 | 22 | 22 | 22 | 23 | 23 | 25 | 25 | 26 | 26 |  |
| MCC (%) | 56.3 | 79.5 | 82.2 | 81.6 | 80.9 | 81.7 | 82.1 | 81.4 | 80.3 | 80.2 | 81.0 | 79.9 | 79.2 | 79.7 | 80.1 | 79.6 | 79.6 | 79.3 | 79.8 | 79.3 | 79.3 |  |
| Sensitivity (%) | 97.7 | 97.7 | 96.9 | 94.5 | 91.4 | 89.8 | 89.8 | 88.3 | 86.7 | 85.9 | 85.9 | 84.4 | 82.8 | 82.8 | 82.8 | 82.0 | 82.0 | 80.5 | 80.5 | 79.7 | 79.7 |  |
| Specificity (%) | 73.0 | 91.1 | 92.9 | 93.5 | 94.2 | 95.1 | 95.3 | 95.5 | 95.5 | 95.6 | 96.0 | 96.0 | 96.2 | 96.4 | 96.6 | 96.6 | 96.6 | 96.9 | 97.1 | 97.1 | 97.1 |  |
| Accuracy (%) | 77.6 | 92.3 | 93.7 | 93.7 | 93.7 | 94.1 | 94.3 | 94.1 | 93.8 | 93.8 | 94.1 | 93.8 | 93.7 | 93.8 | 94.0 | 93.8 | 93.8 | 93.8 | 94.0 | 93.8 | 93.8 |  |
| Balanced Accuracy (%) | 85.4 | 94.4 | 94.9 | 94.0 | 92.8 | 92.4 | 92.6 | 91.9 | 91.1 | 90.8 | 91.0 | 90.2 | 89.5 | 89.6 | 89.7 | 89.3 | 89.3 | 88.7 | 88.8 | 88.4 | 88.4 |  |
| Note. — The thresholds are in mm³. CTPAs = computed tomography (CT) pulmonary angiography (CTPA) examinations, TN = true-negative CTPAs, FP = false-positive CTPAs, TP = true-positive CTPAs, FN = false-negative CTPAs, MCC = Matthew’s correlation coefficient. | | | | | | | | | | | | | | | | | | | | | |  |
|  |  |  |  |  |  |  |  |  |  |  |  |  |  |  |  |  |  |  |  |  |  |  |
|  |  |  |  |  |  |  |  |  |  |  |  |  |  |  |  |  |  |  |  |  |  |  |

***Supplementary Table 5. Diagnostic performance of the trained model with post-processing strategy 1 in the external FUMPE Dataset***

|  | FUMPE External Dataset (CTPAs = 34) | | | | | | | | | | | | | | | | | | | | |  |
| --- | --- | --- | --- | --- | --- | --- | --- | --- | --- | --- | --- | --- | --- | --- | --- | --- | --- | --- | --- | --- | --- | --- |
|  | Test Time Augmentation Enabled | | | | | | | | | | | | | | | | | | | | |  |
| Metric \ Threshold | 0 | 10 | 20 | 30 | 40 | 50 | 60 | 70 | 80 | 90 | 100 | 110 | 120 | 130 | 140 | 150 | 160 | 170 | 180 | 190 | 200 |  |
| No. of TN | 2 | 2 | 2 | 2 | 2 | 2 | 2 | 2 | 2 | 2 | 2 | 2 | 2 | 2 | 2 | 2 | 2 | 2 | 2 | 2 | 2 |  |
| No. of FP | 0 | 0 | 0 | 0 | 0 | 0 | 0 | 0 | 0 | 0 | 0 | 0 | 0 | 0 | 0 | 0 | 0 | 0 | 0 | 0 | 0 |  |
| No. of TP | 31 | 31 | 31 | 31 | 30 | 29 | 29 | 29 | 29 | 28 | 28 | 28 | 28 | 28 | 28 | 28 | 28 | 28 | 28 | 27 | 27 |  |
| No. of FN | 1 | 1 | 1 | 1 | 2 | 3 | 3 | 3 | 3 | 4 | 4 | 4 | 4 | 4 | 4 | 4 | 4 | 4 | 4 | 5 | 5 |  |
| MCC (%) | 80 | 80.4 | 80.4 | 80.4 | 68.5 | 60.2 | 60.2 | 60.2 | 60.2 | 54.0 | 54.0 | 54.0 | 54.0 | 54.0 | 54.0 | 54.0 | 54.0 | 54.0 | 54.0 | 49.1 | 49.1 |  |
| Sensitivity (%) | 97 | 96.9 | 96.9 | 96.9 | 93.8 | 90.6 | 90.6 | 90.6 | 90.6 | 87.5 | 87.5 | 87.5 | 87.5 | 87.5 | 87.5 | 87.5 | 87.5 | 87.5 | 87.5 | 84.4 | 84.4 |  |
| Specificity (%) | 100 | 100 | 100 | 100 | 100 | 100 | 100 | 100 | 100 | 100 | 100 | 100 | 100 | 100 | 100 | 100 | 100 | 100 | 100 | 100 | 100 |  |
| Accuracy (%) | 97 | 97.1 | 97.1 | 97.1 | 94.1 | 91.2 | 91.2 | 91.2 | 91.2 | 88.2 | 88.2 | 88.2 | 88.2 | 88.2 | 88.2 | 88.2 | 88.2 | 88.2 | 88.2 | 85.3 | 85.3 |  |
| Balanced Accuracy (%) | 98 | 98.4 | 98.4 | 98.4 | 96.9 | 95.3 | 95.3 | 95.3 | 95.3 | 93.8 | 93.8 | 93.8 | 93.8 | 93.8 | 93.8 | 93.8 | 93.8 | 93.8 | 93.8 | 92.2 | 92.2 |  |
|  | Test Time Augmentation Disabled | | | | | | | | | | | | | | | | | | | | |  |
| No. of TN | 2 | 2 | 2 | 2 | 2 | 2 | 2 | 2 | 2 | 2 | 2 | 2 | 2 | 2 | 2 | 2 | 2 | 2 | 2 | 2 | 2 |  |
| No. of FP | 0 | 0 | 0 | 0 | 0 | 0 | 0 | 0 | 0 | 0 | 0 | 0 | 0 | 0 | 0 | 0 | 0 | 0 | 0 | 0 | 0 |  |
| No. of TP | 32 | 31 | 31 | 31 | 30 | 30 | 29 | 29 | 29 | 29 | 29 | 29 | 29 | 29 | 29 | 29 | 29 | 29 | 29 | 29 | 28 |  |
| No. of FN | 0 | 1 | 1 | 1 | 2 | 2 | 3 | 3 | 3 | 3 | 3 | 3 | 3 | 3 | 3 | 3 | 3 | 3 | 3 | 3 | 4 |  |
| MCC (%) | 100 | 80.4 | 80.4 | 80.4 | 68.5 | 68.5 | 60.2 | 60.2 | 60.2 | 60.2 | 60.2 | 60.2 | 60.2 | 60.2 | 60.2 | 60.2 | 60.2 | 60.2 | 60.2 | 60.2 | 54.0 |  |
| Sensitivity (%) | 100 | 96.9 | 96.9 | 96.9 | 93.8 | 93.8 | 90.6 | 90.6 | 90.6 | 90.6 | 90.6 | 90.6 | 90.6 | 90.6 | 90.6 | 90.6 | 90.6 | 90.6 | 90.6 | 90.6 | 87.5 |  |
| Specificity (%) | 100 | 100 | 100 | 100 | 100 | 100 | 100 | 100 | 100 | 100 | 100 | 100 | 100 | 100 | 100 | 100 | 100 | 100 | 100 | 100 | 100 |  |
| Accuracy (%) | 100 | 97.1 | 97.1 | 97.1 | 94.1 | 94.1 | 91.2 | 91.2 | 91.2 | 91.2 | 91.2 | 91.2 | 91.2 | 91.2 | 91.2 | 91.2 | 91.2 | 91.2 | 91.2 | 91.2 | 88.2 |  |
| Balanced Accuracy (%) | 100 | 98.4 | 98.4 | 98.4 | 96.9 | 96.9 | 95.3 | 95.3 | 95.3 | 95.3 | 95.3 | 95.3 | 95.3 | 95.3 | 95.3 | 95.3 | 95.3 | 95.3 | 95.3 | 95.3 | 93.8 |  |
| Note. — The thresholds are in mm³. CTPAs = computed tomography (CT) pulmonary angiography (CTPA) examinations, TN = true-negative CTPAs, FP = false-positive CTPAs, TP = true-positive CTPAs, FN = false-negative CTPAs, MCC = Matthew’s correlation coefficient. FUMPE = Ferdowsi University of Mashhad's PE dataset. | | | | | | | | | | | | | | | | | | | | | |  |
|  |  |  |  |  |  |  |  |  |  |  |  |  |  |  |  |  |  |  |  |  |  |  |
|  |  |  |  |  |  |  |  |  |  |  |  |  |  |  |  |  |  |  |  |  |  |  |

***Supplementary Table 6. Diagnostic performance of the trained model with post-processing strategy 1 in the external RSPECT Dataset***

|  | RSPECT External Dataset (CTPAs = 770) | | | | | | | | | | | | | | | | | | | | |  |
| --- | --- | --- | --- | --- | --- | --- | --- | --- | --- | --- | --- | --- | --- | --- | --- | --- | --- | --- | --- | --- | --- | --- |
|  | Test Time Augmentation Enabled | | | | | | | | | | | | | | | | | | | | |  |
| Metric \ Threshold | 0 | 10 | 20 | 30 | 40 | 50 | 60 | 70 | 80 | 90 | 100 | 110 | 120 | 130 | 140 | 150 | 160 | 170 | 180 | 190 | 200 |  |
| No. of TN | 294 | 336 | 346 | 355 | 361 | 364 | 368 | 370 | 370 | 371 | 372 | 374 | 376 | 377 | 377 | 377 | 377 | 378 | 380 | 380 | 380 |  |
| No. of FP | 91 | 49 | 39 | 30 | 24 | 21 | 17 | 15 | 15 | 14 | 13 | 11 | 9 | 8 | 8 | 8 | 8 | 7 | 5 | 5 | 5 |  |
| No. of TP | 382 | 379 | 379 | 379 | 377 | 376 | 373 | 372 | 372 | 371 | 369 | 369 | 368 | 367 | 367 | 366 | 365 | 364 | 364 | 364 | 363 |  |
| No. of FN | 3 | 6 | 6 | 6 | 8 | 9 | 12 | 13 | 13 | 14 | 16 | 16 | 17 | 18 | 18 | 19 | 20 | 21 | 21 | 21 | 22 |  |
| MCC (%) | 77.6 | 86.3 | 88.6 | 90.8 | 91.8 | 92.3 | 92.5 | 92.7 | 92.7 | 92.7 | 92.5 | 93.0 | 93.3 | 93.3 | 93.3 | 93.0 | 92.8 | 92.8 | 93.3 | 93.3 | 93.1 |  |
| Sensitivity (%) | 99.2 | 98.4 | 98.4 | 98.4 | 97.9 | 97.7 | 96.9 | 96.6 | 96.6 | 96.4 | 95.8 | 95.8 | 95.6 | 95.3 | 95.3 | 95.1 | 94.8 | 94.5 | 94.5 | 94.5 | 94.3 |  |
| Specificity (%) | 76.4 | 87.3 | 89.9 | 92.2 | 93.8 | 94.5 | 95.6 | 96.1 | 96.1 | 96.4 | 96.6 | 97.1 | 97.7 | 97.9 | 97.9 | 97.9 | 97.9 | 98.2 | 98.7 | 98.7 | 98.7 |  |
| Accuracy (%) | 87.8 | 92.9 | 94.2 | 95.3 | 95.8 | 96.1 | 96.2 | 96.4 | 96.4 | 96.4 | 96.2 | 96.5 | 96.6 | 96.6 | 96.6 | 96.5 | 96.4 | 96.4 | 96.6 | 96.6 | 96.5 |  |
| Balanced Accuracy (%) | 87.8 | 92.8 | 94.2 | 95.3 | 95.8 | 96.1 | 96.2 | 96.4 | 96.4 | 96.4 | 96.2 | 96.4 | 96.6 | 96.6 | 96.6 | 96.5 | 96.4 | 96.4 | 96.6 | 96.6 | 96.5 |  |
|  | Test Time Augmentation Disabled | | | | | | | | | | | | | | | | | | | | |  |
| No. of TN | 261 | 325 | 339 | 344 | 347 | 356 | 359 | 363 | 364 | 365 | 366 | 370 | 370 | 370 | 371 | 372 | 373 | 375 | 377 | 377 | 377 |  |
| No. of FP | 124 | 60 | 46 | 41 | 38 | 29 | 26 | 22 | 21 | 20 | 19 | 15 | 15 | 15 | 14 | 13 | 12 | 10 | 8 | 8 | 8 |  |
| No. of TP | 381 | 378 | 378 | 378 | 376 | 375 | 373 | 372 | 370 | 369 | 368 | 368 | 367 | 367 | 366 | 364 | 364 | 364 | 364 | 363 | 363 |  |
| No. of FN | 4 | 7 | 7 | 7 | 9 | 10 | 12 | 13 | 15 | 16 | 17 | 17 | 18 | 18 | 19 | 21 | 21 | 21 | 21 | 22 | 22 |  |
| MCC (%) | 70.3 | 83.4 | 86.7 | 87.9 | 88.0 | 90.0 | 90.2 | 90.9 | 90.7 | 90.7 | 90.7 | 91.7 | 91.4 | 91.4 | 91.4 | 91.2 | 91.5 | 92.0 | 92.5 | 92.3 | 92.3 |  |
| Sensitivity (%) | 99.0 | 98.2 | 98.2 | 98.2 | 97.7 | 97.4 | 96.9 | 96.6 | 96.1 | 95.8 | 95.6 | 95.6 | 95.3 | 95.3 | 95.1 | 94.5 | 94.5 | 94.5 | 94.5 | 94.3 | 94.3 |  |
| Specificity (%) | 67.8 | 84.4 | 88.1 | 89.4 | 90.1 | 92.5 | 93.2 | 94.3 | 94.5 | 94.8 | 95.1 | 96.1 | 96.1 | 96.1 | 96.4 | 96.6 | 96.9 | 97.4 | 97.9 | 97.9 | 97.9 |  |
| Accuracy (%) | 83.4 | 91.3 | 93.1 | 93.8 | 93.9 | 94.9 | 95.1 | 95.5 | 95.3 | 95.3 | 95.3 | 95.8 | 95.7 | 95.7 | 95.7 | 95.6 | 95.7 | 96.0 | 96.2 | 96.1 | 96.1 |  |
| Balanced Accuracy (%) | 83.4 | 91.3 | 93.2 | 93.8 | 93.9 | 94.9 | 95.0 | 95.4 | 95.3 | 95.3 | 95.4 | 95.8 | 95.7 | 95.7 | 95.8 | 95.6 | 95.7 | 95.9 | 96.2 | 96.1 | 96.1 |  |
| Note. — The thresholds are in mm³. CTPAs = computed tomography (CT) pulmonary angiography (CTPA) examinations, TN = true-negative CTPAs, FP = false-positive CTPAs, TP = true-positive CTPAs, FN = false-negative CTPAs, MCC = Matthew’s correlation coefficient. RSPECT = RSNA Pulmonary Embolism CT Dataset. | | | | | | | | | | | | | | | | | | | | | |  |
|  |  |  |  |  |  |  |  |  |  |  |  |  |  |  |  |  |  |  |  |  |  |  |
|  |  |  |  |  |  |  |  |  |  |  |  |  |  |  |  |  |  |  |  |  |  |  |

***Supplementary Table 7. Diagnostic performance of the trained model with post-processing strategy 2 in the internal Dataset***

|  | Internal Dataset (CTPAs = 679) | | | | | | | | | | | | | | | | | | | | |  |
| --- | --- | --- | --- | --- | --- | --- | --- | --- | --- | --- | --- | --- | --- | --- | --- | --- | --- | --- | --- | --- | --- | --- |
|  | Test Time Augmentation Enabled | | | | | | | | | | | | | | | | | | | | |  |
| Metric \ Threshold | 0 | 10 | 20 | 30 | 40 | 50 | 60 | 70 | 80 | 90 | 100 | 110 | 120 | 130 | 140 | 150 | 160 | 170 | 180 | 190 | 200 |  |
| No. of TN | 466 | 519 | 524 | 529 | 531 | 533 | 534 | 536 | 536 | 537 | 538 | 538 | 538 | 540 | 540 | 540 | 540 | 540 | 541 | 541 | 541 |  |
| No. of FP | 85 | 32 | 27 | 22 | 20 | 18 | 17 | 15 | 15 | 14 | 13 | 13 | 13 | 11 | 11 | 11 | 11 | 11 | 10 | 10 | 10 |  |
| No. of TP | 124 | 120 | 116 | 114 | 113 | 112 | 109 | 108 | 108 | 107 | 106 | 106 | 106 | 105 | 103 | 102 | 102 | 101 | 100 | 99 | 99 |  |
| No. of FN | 4 | 8 | 12 | 14 | 15 | 16 | 19 | 20 | 20 | 21 | 22 | 22 | 22 | 23 | 25 | 26 | 26 | 27 | 28 | 29 | 29 |  |
| MCC (%) | 69.0 | 82.5 | 82.2 | 83.1 | 83.4 | 83.7 | 82.6 | 82.9 | 82.9 | 82.8 | 82.8 | 82.8 | 82.8 | 83.2 | 82.1 | 81.6 | 81.6 | 81.1 | 81.0 | 80.5 | 80.5 |  |
| Sensitivity (%) | 96.9 | 93.8 | 90.6 | 89.1 | 88.3 | 87.5 | 85.2 | 84.4 | 84.4 | 83.6 | 82.8 | 82.8 | 82.8 | 82.0 | 80.5 | 79.7 | 79.7 | 78.9 | 78.1 | 77.3 | 77.3 |  |
| Specificity (%) | 84.6 | 94.2 | 95.1 | 96.0 | 96.4 | 96.7 | 96.9 | 97.3 | 97.3 | 97.5 | 97.6 | 97.6 | 97.6 | 98.0 | 98.0 | 98.0 | 98.0 | 98.0 | 98.2 | 98.2 | 98.2 |  |
| Accuracy (%) | 86.9 | 94.1 | 94.3 | 94.7 | 94.8 | 95.0 | 94.7 | 94.8 | 94.8 | 94.8 | 94.8 | 94.8 | 94.8 | 95.0 | 94.7 | 94.6 | 94.6 | 94.4 | 94.4 | 94.3 | 94.3 |  |
| Balanced Accuracy (%) | 90.8 | 94.0 | 92.8 | 92.6 | 92.4 | 92.1 | 91.0 | 90.8 | 90.8 | 90.6 | 90.2 | 90.2 | 90.2 | 90.0 | 89.2 | 88.8 | 88.8 | 88.4 | 88.2 | 87.8 | 87.8 |  |
|  | Test Time Augmentation Disabled | | | | | | | | | | | | | | | | | | | | |  |
| No. of TN | 419 | 506 | 516 | 521 | 525 | 525 | 527 | 528 | 529 | 529 | 530 | 533 | 533 | 533 | 534 | 535 | 536 | 536 | 536 | 536 | 536 |  |
| No. of FP | 132 | 45 | 35 | 30 | 26 | 26 | 24 | 23 | 22 | 22 | 21 | 18 | 18 | 18 | 17 | 16 | 15 | 15 | 15 | 15 | 15 |  |
| No. of TP | 125 | 123 | 119 | 118 | 115 | 115 | 112 | 109 | 109 | 108 | 106 | 106 | 106 | 106 | 104 | 103 | 103 | 102 | 101 | 101 | 101 |  |
| No. of FN | 3 | 5 | 9 | 10 | 13 | 13 | 16 | 19 | 19 | 20 | 22 | 22 | 22 | 22 | 24 | 25 | 25 | 26 | 27 | 27 | 27 |  |
| MCC (%) | 59.4 | 79.7 | 80.9 | 82.2 | 82.1 | 82.1 | 81.3 | 80.0 | 80.4 | 79.9 | 79.2 | 80.5 | 80.5 | 80.5 | 79.9 | 79.8 | 80.2 | 79.7 | 79.2 | 79.2 | 79.2 |  |
| Sensitivity (%) | 97.7 | 96.1 | 93.0 | 92.2 | 89.8 | 89.8 | 87.5 | 85.2 | 85.2 | 84.4 | 82.8 | 82.8 | 82.8 | 82.8 | 81.2 | 80.5 | 80.5 | 79.7 | 78.9 | 78.9 | 78.9 |  |
| Specificity (%) | 76.0 | 91.8 | 93.6 | 94.6 | 95.3 | 95.3 | 95.6 | 95.8 | 96.0 | 96.0 | 96.2 | 96.7 | 96.7 | 96.7 | 96.9 | 97.1 | 97.3 | 97.3 | 97.3 | 97.3 | 97.3 |  |
| Accuracy (%) | 80.1 | 92.6 | 93.5 | 94.1 | 94.3 | 94.3 | 94.1 | 93.8 | 94.0 | 93.8 | 93.7 | 94.1 | 94.1 | 94.1 | 94.0 | 94.0 | 94.1 | 94.0 | 93.8 | 93.8 | 93.8 |  |
| Balanced Accuracy (%) | 86.8 | 93.9 | 93.3 | 93.4 | 92.6 | 92.6 | 91.6 | 90.5 | 90.6 | 90.2 | 89.5 | 89.8 | 89.8 | 89.8 | 89.0 | 88.8 | 88.9 | 88.5 | 88.1 | 88.1 | 88.1 |  |
| Note. — The thresholds are in mm³. CTPAs = computed tomography (CT) pulmonary angiography (CTPA) examinations, TN = true-negative CTPAs, FP = false-positive CTPAs, TP = true-positive CTPAs, FN = false-negative CTPAs, MCC = Matthew’s correlation coefficient. | | | | | | | | | | | | | | | | | | | | | |  |
|  |  |  |  |  |  |  |  |  |  |  |  |  |  |  |  |  |  |  |  |  |  |  |
|  |  |  |  |  |  |  |  |  |  |  |  |  |  |  |  |  |  |  |  |  |  |  |

***Supplementary Table 8. Diagnostic performance of the trained model with post-processing strategy 2 in the eternal FUMPE Dataset***

|  | FUMPE External Dataset (CTPAs = 34) | | | | | | | | | | | | | | | | | | | | |  |
| --- | --- | --- | --- | --- | --- | --- | --- | --- | --- | --- | --- | --- | --- | --- | --- | --- | --- | --- | --- | --- | --- | --- |
|  | Test Time Augmentation Enabled | | | | | | | | | | | | | | | | | | | | |  |
| Metric \ Threshold | 0 | 10 | 20 | 30 | 40 | 50 | 60 | 70 | 80 | 90 | 100 | 110 | 120 | 130 | 140 | 150 | 160 | 170 | 180 | 190 | 200 |  |
| No. of TN | 2 | 2 | 2 | 2 | 2 | 2 | 2 | 2 | 2 | 2 | 2 | 2 | 2 | 2 | 2 | 2 | 2 | 2 | 2 | 2 | 2 |  |
| No. of FP | 0 | 0 | 0 | 0 | 0 | 0 | 0 | 0 | 0 | 0 | 0 | 0 | 0 | 0 | 0 | 0 | 0 | 0 | 0 | 0 | 0 |  |
| No. of TP | 31 | 31 | 31 | 31 | 29 | 29 | 29 | 28 | 28 | 28 | 28 | 28 | 28 | 28 | 28 | 27 | 27 | 27 | 27 | 27 | 27 |  |
| No. of FN | 1 | 1 | 1 | 1 | 3 | 3 | 3 | 4 | 4 | 4 | 4 | 4 | 4 | 4 | 4 | 5 | 5 | 5 | 5 | 5 | 5 |  |
| MCC (%) | 80.4 | 80.4 | 80.4 | 80.4 | 60.2 | 60.2 | 60.2 | 54.0 | 54.0 | 54.0 | 54.0 | 54.0 | 54.0 | 54.0 | 54.0 | 49.1 | 49.1 | 49.1 | 49.1 | 49.1 | 49.1 |  |
| Sensitivity (%) | 96.9 | 96.9 | 96.9 | 96.9 | 90.6 | 90.6 | 90.6 | 87.5 | 87.5 | 87.5 | 87.5 | 87.5 | 87.5 | 87.5 | 87.5 | 84.4 | 84.4 | 84.4 | 84.4 | 84.4 | 84.4 |  |
| Specificity (%) | 100 | 100 | 100 | 100 | 100 | 100 | 100 | 100 | 100 | 100 | 100 | 100 | 100 | 100 | 100 | 100 | 100 | 100 | 100 | 100 | 100 |  |
| Accuracy (%) | 97.1 | 97.1 | 97.1 | 97.1 | 91.2 | 91.2 | 91.2 | 88.2 | 88.2 | 88.2 | 88.2 | 88.2 | 88.2 | 88.2 | 88.2 | 85.3 | 85.3 | 85.3 | 85.3 | 85.3 | 85.3 |  |
| Balanced Accuracy (%) | 98.4 | 98.4 | 98.4 | 98.4 | 95.3 | 95.3 | 95.3 | 93.8 | 93.8 | 93.8 | 93.8 | 93.8 | 93.8 | 93.8 | 93.8 | 92.2 | 92.2 | 92.2 | 92.2 | 92.2 | 92.2 |  |
|  | Test Time Augmentation Disabled | | | | | | | | | | | | | | | | | | | | |  |
| No. of TN | 2 | 2 | 2 | 2 | 2 | 2 | 2 | 2 | 2 | 2 | 2 | 2 | 2 | 2 | 2 | 2 | 2 | 2 | 2 | 2 | 2 |  |
| No. of FP | 0 | 0 | 0 | 0 | 0 | 0 | 0 | 0 | 0 | 0 | 0 | 0 | 0 | 0 | 0 | 0 | 0 | 0 | 0 | 0 | 0 |  |
| No. of TP | 31 | 31 | 31 | 30 | 30 | 29 | 29 | 29 | 29 | 29 | 29 | 29 | 29 | 29 | 29 | 29 | 28 | 28 | 28 | 28 | 28 |  |
| No. of FN | 1 | 1 | 1 | 2 | 2 | 3 | 3 | 3 | 3 | 3 | 3 | 3 | 3 | 3 | 3 | 3 | 4 | 4 | 4 | 4 | 4 |  |
| MCC (%) | 80.4 | 80.4 | 80.4 | 68.5 | 68.5 | 60.2 | 60.2 | 60.2 | 60.2 | 60.2 | 60.2 | 60.2 | 60.2 | 60.2 | 60.2 | 60.2 | 54.0 | 54.0 | 54.0 | 54.0 | 54.0 |  |
| Sensitivity (%) | 96.9 | 96.9 | 96.9 | 93.8 | 93.8 | 90.6 | 90.6 | 90.6 | 90.6 | 90.6 | 90.6 | 90.6 | 90.6 | 90.6 | 90.6 | 90.6 | 87.5 | 87.5 | 87.5 | 87.5 | 87.5 |  |
| Specificity (%) | 100 | 100 | 100 | 100 | 100 | 100 | 100 | 100 | 100 | 100 | 100 | 100 | 100 | 100 | 100 | 100 | 100 | 100 | 100 | 100 | 100 |  |
| Accuracy (%) | 97.1 | 97.1 | 97.1 | 94.1 | 94.1 | 91.2 | 91.2 | 91.2 | 91.2 | 91.2 | 91.2 | 91.2 | 91.2 | 91.2 | 91.2 | 91.2 | 88.2 | 88.2 | 88.2 | 88.2 | 88.2 |  |
| Balanced Accuracy (%) | 98.4 | 98.4 | 98.4 | 96.9 | 96.9 | 95.3 | 95.3 | 95.3 | 95.3 | 95.3 | 95.3 | 95.3 | 95.3 | 95.3 | 95.3 | 95.3 | 93.8 | 93.8 | 93.8 | 93.8 | 93.8 |  |
| Note. — The thresholds are in mm³. CTPAs = computed tomography (CT) pulmonary angiography (CTPA) examinations, TN = true-negative CTPAs, FP = false-positive CTPAs, TP = true-positive CTPAs, FN = false-negative CTPAs, MCC = Matthew’s correlation coefficient. FUMPE = Ferdowsi University of Mashhad's PE dataset. | | | | | | | | | | | | | | | | | | | | | |  |
|  |  |  |  |  |  |  |  |  |  |  |  |  |  |  |  |  |  |  |  |  |  |  |
|  |  |  |  |  |  |  |  |  |  |  |  |  |  |  |  |  |  |  |  |  |  |  |

***Supplementary Table 9. Diagnostic performance of the trained model with post-processing strategy 2 in the external RSPECT Dataset***

|  | RSPECT External Dataset (CTPAs = 770) | | | | | | | | | | | | | | | | | | | | |  |
| --- | --- | --- | --- | --- | --- | --- | --- | --- | --- | --- | --- | --- | --- | --- | --- | --- | --- | --- | --- | --- | --- | --- |
|  | Test Time Augmentation Enabled | | | | | | | | | | | | | | | | | | | | |  |
| Metric \ Threshold | 0 | 10 | 20 | 30 | 40 | 50 | 60 | 70 | 80 | 90 | 100 | 110 | 120 | 130 | 140 | 150 | 160 | 170 | 180 | 190 | 200 |  |
| No. of TN | 310 | 349 | 358 | 366 | 371 | 373 | 373 | 375 | 376 | 377 | 379 | 379 | 379 | 380 | 381 | 382 | 382 | 382 | 382 | 382 | 382 |  |
| No. of FP | 75 | 36 | 27 | 19 | 14 | 12 | 12 | 10 | 9 | 8 | 6 | 6 | 6 | 5 | 4 | 3 | 3 | 3 | 3 | 3 | 3 |  |
| No. of TP | 380 | 378 | 374 | 374 | 372 | 372 | 370 | 369 | 367 | 366 | 366 | 365 | 364 | 362 | 362 | 362 | 362 | 362 | 362 | 361 | 360 |  |
| No. of FN | 5 | 7 | 11 | 11 | 13 | 13 | 15 | 16 | 18 | 19 | 19 | 20 | 21 | 23 | 23 | 23 | 23 | 23 | 23 | 24 | 25 |  |
| MCC (%) | 80.6 | 89.1 | 90.2 | 92.2 | 93.0 | 93.5 | 93.0 | 93.3 | 93.0 | 93.0 | 93.6 | 93.3 | 93.1 | 92.8 | 93.1 | 93.4 | 93.4 | 93.4 | 93.4 | 93.1 | 92.9 |  |
| Sensitivity (%) | 98.7 | 98.2 | 97.1 | 97.1 | 96.6 | 96.6 | 96.1 | 95.8 | 95.3 | 95.1 | 95.1 | 94.8 | 94.5 | 94.0 | 94.0 | 94.0 | 94.0 | 94.0 | 94.0 | 93.8 | 93.5 |  |
| Specificity (%) | 80.5 | 90.6 | 93.0 | 95.1 | 96.4 | 96.9 | 96.9 | 97.4 | 97.7 | 97.9 | 98.4 | 98.4 | 98.4 | 98.7 | 99.0 | 99.2 | 99.2 | 99.2 | 99.2 | 99.2 | 99.2 |  |
| Accuracy (%) | 89.6 | 94.4 | 95.1 | 96.1 | 96.5 | 96.8 | 96.5 | 96.6 | 96.5 | 96.5 | 96.8 | 96.6 | 96.5 | 96.4 | 96.5 | 96.6 | 96.6 | 96.6 | 96.6 | 96.5 | 96.4 |  |
| Balanced Accuracy (%) | 89.6 | 94.4 | 95.0 | 96.1 | 96.5 | 96.8 | 96.5 | 96.6 | 96.5 | 96.5 | 96.8 | 96.6 | 96.4 | 96.4 | 96.5 | 96.6 | 96.6 | 96.6 | 96.6 | 96.5 | 96.4 |  |
|  | Test Time Augmentation Disabled | | | | | | | | | | | | | | | | | | | | |  |
| No. of TN | 286 | 336 | 347 | 355 | 362 | 365 | 367 | 369 | 370 | 371 | 371 | 372 | 374 | 375 | 376 | 377 | 378 | 378 | 379 | 379 | 381 |  |
| No. of FP | 99 | 49 | 38 | 30 | 23 | 20 | 18 | 16 | 15 | 14 | 14 | 13 | 11 | 10 | 9 | 8 | 7 | 7 | 6 | 6 | 4 |  |
| No. of TP | 381 | 378 | 378 | 376 | 371 | 371 | 368 | 368 | 367 | 367 | 366 | 365 | 364 | 363 | 363 | 362 | 362 | 362 | 362 | 362 | 361 |  |
| No. of FN | 4 | 7 | 7 | 9 | 14 | 14 | 17 | 17 | 18 | 18 | 19 | 20 | 21 | 22 | 22 | 23 | 23 | 23 | 23 | 23 | 24 |  |
| MCC (%) | 75.6 | 86.0 | 88.6 | 90.0 | 90.4 | 91.2 | 90.9 | 91.4 | 91.4 | 91.7 | 91.4 | 91.4 | 91.7 | 91.7 | 92.0 | 92.0 | 92.3 | 92.3 | 92.6 | 92.6 | 92.9 |  |
| Sensitivity (%) | 99.0 | 98.2 | 98.2 | 97.7 | 96.4 | 96.4 | 95.6 | 95.6 | 95.3 | 95.3 | 95.1 | 94.8 | 94.5 | 94.3 | 94.3 | 94.0 | 94.0 | 94.0 | 94.0 | 94.0 | 93.8 |  |
| Specificity (%) | 74.3 | 87.3 | 90.1 | 92.2 | 94.0 | 94.8 | 95.3 | 95.8 | 96.1 | 96.4 | 96.4 | 96.6 | 97.1 | 97.4 | 97.7 | 97.9 | 98.2 | 98.2 | 98.4 | 98.4 | 99.0 |  |
| Accuracy (%) | 86.6 | 92.7 | 94.2 | 94.9 | 95.2 | 95.6 | 95.5 | 95.7 | 95.7 | 95.8 | 95.7 | 95.7 | 95.8 | 95.8 | 96.0 | 96.0 | 96.1 | 96.1 | 96.2 | 96.2 | 96.4 |  |
| Balanced Accuracy (%) | 86.6 | 92.8 | 94.2 | 95.0 | 95.2 | 95.6 | 95.4 | 95.7 | 95.7 | 95.8 | 95.8 | 95.7 | 95.8 | 95.8 | 96.0 | 95.9 | 96.1 | 96.1 | 96.2 | 96.2 | 96.4 |  |
| Note. — The thresholds are in mm³. CTPAs = computed tomography (CT) pulmonary angiography (CTPA) examinations, TN = true-negative CTPAs, FP = false-positive CTPAs, TP = true-positive CTPAs, FN = false-negative CTPAs, MCC = Matthew’s correlation coefficient. RSPECT = RSNA Pulmonary Embolism CT Dataset. | | | | | | | | | | | | | | | | | | | | | |  |
|  |  |  |  |  |  |  |  |  |  |  |  |  |  |  |  |  |  |  |  |  |  |  |
|  |  |  |  |  |  |  |  |  |  |  |  |  |  |  |  |  |  |  |  |  |  |  |

***Supplementary Table 10. Diagnostic performance of the trained model without post-processing strategy in the combined testing dataset***

|  | Testing Dataset (CTPAs = 1355) | | | | | | | | | | | | | | | | | | | | |  |
| --- | --- | --- | --- | --- | --- | --- | --- | --- | --- | --- | --- | --- | --- | --- | --- | --- | --- | --- | --- | --- | --- | --- |
|  | Test Time Augmentation Enabled | | | | | | | | | | | | | | | | | | | | |  |
| Metric \ Threshold | 0 | 10 | 20 | 30 | 40 | 50 | 60 | 70 | 80 | 90 | 100 | 110 | 120 | 130 | 140 | 150 | 160 | 170 | 180 | 190 | 200 |  |
| No. of TN | 346 | 640 | 704 | 748 | 764 | 781 | 796 | 802 | 806 | 812 | 816 | 824 | 827 | 831 | 836 | 836 | 839 | 841 | 842 | 846 | 848 |  |
| No. of FP | 592 | 298 | 234 | 190 | 174 | 157 | 142 | 136 | 132 | 126 | 122 | 114 | 111 | 107 | 102 | 102 | 99 | 97 | 96 | 92 | 90 |  |
| No. of TP | 417 | 417 | 417 | 417 | 417 | 417 | 416 | 415 | 414 | 414 | 413 | 413 | 412 | 410 | 405 | 405 | 405 | 404 | 403 | 403 | 403 |  |
| No. of FN | 0 | 0 | 0 | 0 | 0 | 0 | 1 | 2 | 3 | 3 | 4 | 4 | 5 | 7 | 12 | 12 | 12 | 13 | 14 | 14 | 14 |  |
| MCC (%) | 39.0 | 63.1 | 69.3 | 74.0 | 75.8 | 77.8 | 79.4 | 79.9 | 80.2 | 80.9 | 81.2 | 82.3 | 82.5 | 82.6 | 82.3 | 82.3 | 82.7 | 82.7 | 82.7 | 83.2 | 83.5 |  |
| Sensitivity (%) | 100 | 100 | 100 | 100 | 100 | 100 | 99.8 | 99.5 | 99.3 | 99.3 | 99.0 | 99.0 | 98.8 | 98.3 | 97.1 | 97.1 | 97.1 | 96.9 | 96.6 | 96.6 | 96.6 |  |
| Specificity (%) | 36.9 | 68.2 | 75.1 | 79.7 | 81.4 | 83.3 | 84.9 | 85.5 | 85.9 | 86.6 | 87.0 | 87.8 | 88.2 | 88.6 | 89.1 | 89.1 | 89.4 | 89.7 | 89.8 | 90.2 | 90.4 |  |
| Accuracy (%) | 56.3 | 78.0 | 82.7 | 86.0 | 87.2 | 88.4 | 89.4 | 89.8 | 90.0 | 90.5 | 90.7 | 91.3 | 91.4 | 91.6 | 91.6 | 91.6 | 91.8 | 91.9 | 91.9 | 92.2 | 92.3 |  |
| Balanced Accuracy (%) | 68.4 | 84.1 | 87.6 | 89.8 | 90.7 | 91.6 | 92.4 | 92.5 | 92.6 | 92.9 | 93.0 | 93.4 | 93.5 | 93.4 | 93.1 | 93.1 | 93.2 | 93.3 | 93.2 | 93.4 | 93.5 |  |
|  | Test Time Augmentation Disabled | | | | | | | | | | | | | | | | | | | | |  |
| No. of TN | 308 | 615 | 680 | 718 | 740 | 757 | 779 | 786 | 794 | 804 | 809 | 812 | 819 | 824 | 827 | 829 | 832 | 838 | 840 | 843 | 843 |  |
| No. of FP | 630 | 323 | 258 | 220 | 198 | 181 | 159 | 152 | 144 | 134 | 129 | 126 | 119 | 114 | 111 | 109 | 106 | 100 | 98 | 95 | 95 |  |
| No. of TP | 417 | 417 | 417 | 417 | 417 | 416 | 416 | 415 | 415 | 415 | 415 | 415 | 414 | 414 | 411 | 409 | 407 | 406 | 406 | 406 | 406 |  |
| No. of FN | 0 | 0 | 0 | 0 | 0 | 1 | 1 | 2 | 2 | 2 | 2 | 2 | 3 | 3 | 6 | 8 | 10 | 11 | 11 | 11 | 11 |  |
| MCC (%) | 36.2 | 60.8 | 66.9 | 70.8 | 73.1 | 74.8 | 77.3 | 78.0 | 78.9 | 80.1 | 80.8 | 81.1 | 81.8 | 82.5 | 82.3 | 82.1 | 82.1 | 82.7 | 83.0 | 83.4 | 83.4 |  |
| Sensitivity (%) | 100.0 | 100.0 | 100.0 | 100.0 | 100.0 | 99.8 | 99.8 | 99.5 | 99.5 | 99.5 | 99.5 | 99.5 | 99.3 | 99.3 | 98.6 | 98.1 | 97.6 | 97.4 | 97.4 | 97.4 | 97.4 |  |
| Specificity (%) | 32.8 | 65.6 | 72.5 | 76.5 | 78.9 | 80.7 | 83.0 | 83.8 | 84.6 | 85.7 | 86.2 | 86.6 | 87.3 | 87.8 | 88.2 | 88.4 | 88.7 | 89.3 | 89.6 | 89.9 | 89.9 |  |
| Accuracy (%) | 53.5 | 76.2 | 81.0 | 83.8 | 85.4 | 86.6 | 88.2 | 88.6 | 89.2 | 90.0 | 90.3 | 90.6 | 91.0 | 91.4 | 91.4 | 91.4 | 91.4 | 91.8 | 92.0 | 92.2 | 92.2 |  |
| Balanced Accuracy (%) | 66.4 | 82.8 | 86.2 | 88.2 | 89.4 | 90.2 | 91.4 | 91.6 | 92.0 | 92.6 | 92.8 | 93.0 | 93.3 | 93.6 | 93.4 | 93.2 | 93.2 | 93.4 | 93.5 | 93.6 | 93.6 |  |
| Note. — The thresholds are in mm³. CTPAs = computed tomography (CT) pulmonary angiography (CTPA) examinations, TN = true-negative CTPAs, FP = false-positive CTPAs, TP = true-positive CTPAs, FN = false-negative CTPAs, MCC = Matthew’s correlation coefficient. RSPECT = RSNA Pulmonary Embolism CT Dataset, FUMPE = Ferdowsi University of Mashhad's PE dataset, Testing Dataset = 551 PE negative CTPAs from internal testing set + 32 PE positive and 2 PE negative from FUMPE + 385 PE positive and 385 PE negative CTPAs from RSPECT. | | | | | | | | | | | | | | | | | | | | | |  |
|  |  |  |  |  |  |  |  |  |  |  |  |  |  |  |  |  |  |  |  |  |  |  |
|  |  |  |  |  |  |  |  |  |  |  |  |  |  |  |  |  |  |  |  |  |  |  |

***Supplementary Table 11. Diagnostic performance of the trained model with post-processing strategy 1 in the combined testing dataset***

|  | Testing Dataset (CTPAs = 1355) | | | | | | | | | | | | | | | | | | | | |  |
| --- | --- | --- | --- | --- | --- | --- | --- | --- | --- | --- | --- | --- | --- | --- | --- | --- | --- | --- | --- | --- | --- | --- |
|  | Test Time Augmentation Enabled | | | | | | | | | | | | | | | | | | | | |  |
| Metric \ Threshold | 0 | 10 | 20 | 30 | 40 | 50 | 60 | 70 | 80 | 90 | 100 | 110 | 120 | 130 | 140 | 150 | 160 | 170 | 180 | 190 | 200 |  |
| No. of TN | 735 | 849 | 869 | 880 | 889 | 895 | 899 | 902 | 902 | 903 | 905 | 909 | 912 | 914 | 915 | 915 | 916 | 918 | 920 | 920 | 920 |  |
| No. of FP | 203 | 89 | 69 | 58 | 49 | 43 | 39 | 36 | 36 | 35 | 33 | 29 | 26 | 24 | 23 | 23 | 22 | 20 | 18 | 18 | 18 |  |
| No. of TP | 413 | 410 | 410 | 410 | 407 | 405 | 402 | 401 | 401 | 399 | 397 | 397 | 396 | 395 | 395 | 394 | 393 | 392 | 392 | 391 | 390 |  |
| No. of FN | 4 | 7 | 7 | 7 | 10 | 12 | 15 | 16 | 16 | 18 | 20 | 20 | 21 | 22 | 22 | 23 | 24 | 25 | 25 | 26 | 27 |  |
| MCC (%) | 71.7 | 85.0 | 87.8 | 89.4 | 90.2 | 90.8 | 90.9 | 91.2 | 91.2 | 91.0 | 90.9 | 91.6 | 91.9 | 92.0 | 92.2 | 92.0 | 92.0 | 92.2 | 92.5 | 92.3 | 92.2 |  |
| Sensitivity (%) | 99.0 | 98.3 | 98.3 | 98.3 | 97.6 | 97.1 | 96.4 | 96.2 | 96.2 | 95.7 | 95.2 | 95.2 | 95.0 | 94.7 | 94.7 | 94.5 | 94.2 | 94.0 | 94.0 | 93.8 | 93.5 |  |
| Specificity (%) | 78.4 | 90.5 | 92.6 | 93.8 | 94.8 | 95.4 | 95.8 | 96.2 | 96.2 | 96.3 | 96.5 | 96.9 | 97.2 | 97.4 | 97.5 | 97.5 | 97.7 | 97.9 | 98.1 | 98.1 | 98.1 |  |
| Accuracy (%) | 84.7 | 92.9 | 94.4 | 95.2 | 95.6 | 95.9 | 96.0 | 96.2 | 96.2 | 96.1 | 96.1 | 96.4 | 96.5 | 96.6 | 96.7 | 96.6 | 96.6 | 96.7 | 96.8 | 96.8 | 96.7 |  |
| Balanced Accuracy (%) | 88.7 | 94.4 | 95.4 | 96.0 | 96.2 | 96.2 | 96.1 | 96.2 | 96.2 | 96.0 | 95.8 | 96.0 | 96.1 | 96.0 | 96.1 | 96.0 | 95.9 | 95.9 | 96.0 | 95.9 | 95.8 |  |
|  | Test Time Augmentation Disabled | | | | | | | | | | | | | | | | | | | | |  |
| No. of TN | 665 | 829 | 853 | 861 | 868 | 882 | 886 | 891 | 892 | 894 | 897 | 901 | 902 | 903 | 905 | 906 | 907 | 911 | 914 | 914 | 914 |  |
| No. of FP | 273 | 109 | 85 | 77 | 70 | 56 | 52 | 47 | 46 | 44 | 41 | 37 | 36 | 35 | 33 | 32 | 31 | 27 | 24 | 24 | 24 |  |
| No. of TP | 413 | 409 | 409 | 409 | 406 | 405 | 402 | 401 | 399 | 398 | 397 | 397 | 396 | 396 | 395 | 393 | 393 | 393 | 393 | 392 | 391 |  |
| No. of FN | 4 | 8 | 8 | 8 | 11 | 12 | 15 | 16 | 18 | 19 | 20 | 20 | 21 | 21 | 22 | 24 | 24 | 24 | 24 | 25 | 26 |  |
| MCC (%) | 64.6 | 82.1 | 85.4 | 86.5 | 86.9 | 88.8 | 88.8 | 89.4 | 89.2 | 89.3 | 89.6 | 90.3 | 90.3 | 90.4 | 90.6 | 90.4 | 90.5 | 91.2 | 91.7 | 91.5 | 91.3 |  |
| Sensitivity (%) | 99.0 | 98.1 | 98.1 | 98.1 | 97.4 | 97.1 | 96.4 | 96.2 | 95.7 | 95.4 | 95.2 | 95.2 | 95.0 | 95.0 | 94.7 | 94.2 | 94.2 | 94.2 | 94.2 | 94.0 | 93.8 |  |
| Specificity (%) | 70.9 | 88.4 | 90.9 | 91.8 | 92.5 | 94.0 | 94.5 | 95.0 | 95.1 | 95.3 | 95.6 | 96.1 | 96.2 | 96.3 | 96.5 | 96.6 | 96.7 | 97.1 | 97.4 | 97.4 | 97.4 |  |
| Accuracy (%) | 79.6 | 91.4 | 93.1 | 93.7 | 94.0 | 95.0 | 95.1 | 95.4 | 95.3 | 95.4 | 95.5 | 95.8 | 95.8 | 95.9 | 95.9 | 95.9 | 95.9 | 96.2 | 96.5 | 96.4 | 96.3 |  |
| Balanced Accuracy (%) | 84.9 | 93.2 | 94.5 | 94.9 | 94.9 | 95.6 | 95.4 | 95.6 | 95.4 | 95.4 | 95.4 | 95.6 | 95.6 | 95.6 | 95.6 | 95.4 | 95.4 | 95.6 | 95.8 | 95.7 | 95.6 |  |
| Note. — The thresholds are in mm³. CTPAs = computed tomography (CT) pulmonary angiography (CTPA) examinations, TN = true-negative CTPAs, FP = false-positive CTPAs, TP = true-positive CTPAs, FN = false-negative CTPAs, MCC = Matthew’s correlation coefficient. RSPECT = RSNA Pulmonary Embolism CT Dataset, FUMPE = Ferdowsi University of Mashhad's PE dataset, Testing Dataset = 551 PE negative CTPAs from internal testing set + 32 PE positive and 2 PE negative from FUMPE + 385 PE positive and 385 PE negative CTPAs from RSPECT. | | | | | | | | | | | | | | | | | | | | | |  |
|  |  |  |  |  |  |  |  |  |  |  |  |  |  |  |  |  |  |  |  |  |  |  |
|  |  |  |  |  |  |  |  |  |  |  |  |  |  |  |  |  |  |  |  |  |  |  |

***Supplementary Table 12. Diagnostic performance of the trained model with post-processing strategy 2 in the combined testing dataset***

|  | Testing Dataset (CTPAs = 1355) | | | | | | | | | | | | | | | | | | | | |  |
| --- | --- | --- | --- | --- | --- | --- | --- | --- | --- | --- | --- | --- | --- | --- | --- | --- | --- | --- | --- | --- | --- | --- |
|  | Test Time Augmentation Enabled | | | | | | | | | | | | | | | | | | | | |  |
| Metric \ Threshold | 0 | 10 | 20 | 30 | 40 | 50 | 60 | 70 | 80 | 90 | 100 | 110 | 120 | 130 | 140 | 150 | 160 | 170 | 180 | 190 | 200 |  |
| No. of TN | 778 | 870 | 884 | 897 | 904 | 908 | 909 | 913 | 914 | 916 | 919 | 919 | 919 | 922 | 923 | 924 | 924 | 924 | 925 | 925 | 925 |  |
| No. of FP | 160 | 68 | 54 | 41 | 34 | 30 | 29 | 25 | 24 | 22 | 19 | 19 | 19 | 16 | 15 | 14 | 14 | 14 | 13 | 13 | 13 |  |
| No. of TP | 411 | 409 | 405 | 405 | 401 | 401 | 399 | 397 | 395 | 394 | 394 | 393 | 392 | 390 | 390 | 389 | 389 | 389 | 389 | 388 | 387 |  |
| No. of FN | 6 | 8 | 12 | 12 | 16 | 16 | 18 | 20 | 22 | 23 | 23 | 24 | 25 | 27 | 27 | 28 | 28 | 28 | 28 | 29 | 30 |  |
| MCC (%) | 76.2 | 87.8 | 89.1 | 91.1 | 91.5 | 92.1 | 91.9 | 92.2 | 92.0 | 92.2 | 92.7 | 92.5 | 92.4 | 92.5 | 92.7 | 92.7 | 92.7 | 92.7 | 92.9 | 92.7 | 92.5 |  |
| Sensitivity (%) | 98.6 | 98.1 | 97.1 | 97.1 | 96.2 | 96.2 | 95.7 | 95.2 | 94.7 | 94.5 | 94.5 | 94.2 | 94.0 | 93.5 | 93.5 | 93.3 | 93.3 | 93.3 | 93.3 | 93.0 | 92.8 |  |
| Specificity (%) | 82.9 | 92.8 | 94.2 | 95.6 | 96.4 | 96.8 | 96.9 | 97.3 | 97.4 | 97.7 | 98.0 | 98.0 | 98.0 | 98.3 | 98.4 | 98.5 | 98.5 | 98.5 | 98.6 | 98.6 | 98.6 |  |
| Accuracy (%) | 87.7 | 94.4 | 95.1 | 96.1 | 96.3 | 96.6 | 96.5 | 96.7 | 96.6 | 96.7 | 96.9 | 96.8 | 96.8 | 96.8 | 96.9 | 96.9 | 96.9 | 96.9 | 97.0 | 96.9 | 96.8 |  |
| Balanced Accuracy (%) | 90.8 | 95.4 | 95.6 | 96.4 | 96.3 | 96.5 | 96.3 | 96.2 | 96.0 | 96.1 | 96.2 | 96.1 | 96.0 | 95.9 | 96.0 | 95.9 | 95.9 | 95.9 | 96.0 | 95.8 | 95.7 |  |
|  | Test Time Augmentation Disabled | | | | | | | | | | | | | | | | | | | | |  |
| No. of TN | 707 | 844 | 865 | 878 | 889 | 892 | 896 | 899 | 901 | 902 | 903 | 907 | 909 | 910 | 912 | 914 | 916 | 916 | 917 | 917 | 919 |  |
| No. of FP | 231 | 94 | 73 | 60 | 49 | 46 | 42 | 39 | 37 | 36 | 35 | 31 | 29 | 28 | 26 | 24 | 22 | 22 | 21 | 21 | 19 |  |
| No. of TP | 412 | 409 | 409 | 406 | 401 | 400 | 397 | 397 | 396 | 396 | 395 | 394 | 393 | 392 | 392 | 391 | 390 | 390 | 390 | 390 | 389 |  |
| No. of FN | 5 | 8 | 8 | 11 | 16 | 17 | 20 | 20 | 21 | 21 | 22 | 23 | 24 | 25 | 25 | 26 | 27 | 27 | 27 | 27 | 28 |  |
| MCC (%) | 68.6 | 84.1 | 87.1 | 88.4 | 89.1 | 89.4 | 89.5 | 90.0 | 90.1 | 90.3 | 90.2 | 90.7 | 90.9 | 90.8 | 91.2 | 91.3 | 91.5 | 91.5 | 91.7 | 91.7 | 91.8 |  |
| Sensitivity (%) | 98.8 | 98.1 | 98.1 | 97.4 | 96.2 | 95.9 | 95.2 | 95.2 | 95.0 | 95.0 | 94.7 | 94.5 | 94.2 | 94.0 | 94.0 | 93.8 | 93.5 | 93.5 | 93.5 | 93.5 | 93.3 |  |
| Specificity (%) | 75.4 | 90.0 | 92.2 | 93.6 | 94.8 | 95.1 | 95.5 | 95.8 | 96.1 | 96.2 | 96.3 | 96.7 | 96.9 | 97.0 | 97.2 | 97.4 | 97.7 | 97.7 | 97.8 | 97.8 | 98.0 |  |
| Accuracy (%) | 82.6 | 92.5 | 94.0 | 94.8 | 95.2 | 95.4 | 95.4 | 95.6 | 95.7 | 95.8 | 95.8 | 96.0 | 96.1 | 96.1 | 96.2 | 96.3 | 96.4 | 96.4 | 96.5 | 96.5 | 96.5 |  |
| Balanced Accuracy (%) | 87.1 | 94.0 | 95.2 | 95.5 | 95.5 | 95.5 | 95.4 | 95.5 | 95.6 | 95.6 | 95.5 | 95.6 | 95.5 | 95.5 | 95.6 | 95.6 | 95.6 | 95.6 | 95.6 | 95.6 | 95.6 |  |
| Note. — The thresholds are in mm³. CTPAs = computed tomography (CT) pulmonary angiography (CTPA) examinations, TN = true-negative CTPAs, FP = false-positive CTPAs, TP = true-positive CTPAs, FN = false-negative CTPAs, MCC = Matthew’s correlation coefficient. RSPECT = RSNA Pulmonary Embolism CT Dataset, FUMPE = Ferdowsi University of Mashhad's PE dataset, Testing Dataset = 551 PE negative CTPAs from internal testing set + 32 PE positive and 2 PE negative from FUMPE + 385 PE positive and 385 PE negative CTPAs from RSPECT. | | | | | | | | | | | | | | | | | | | | | |  |
|  |  |  |  |  |  |  |  |  |  |  |  |  |  |  |  |  |  |  |  |  |  |  |
|  |  |  |  |  |  |  |  |  |  |  |  |  |  |  |  |  |  |  |  |  |  |  |
